# Macrophages modulate adult zebrafish tail fin regeneration

**DOI:** 10.1242/dev.098459

**Published:** 2014-07

**Authors:** Timothy A. Petrie, Nicholas S. Strand, Chao Tsung-Yang, Jeremy S. Rabinowitz, Randall T. Moon

**Affiliations:** 1 HHMI, Chevy Chase, MD 20815, USA; 2 Department of Pharmacology, University of Washington, Seattle, WA 98109, USA; 3 Department of Microbiology, University of Washington, Seattle, WA 98105, USA

**Keywords:** Regeneration, Inflammation, Zebrafish, Fin, Macrophages, Neutrophils, Wnt

## Abstract

Neutrophils and macrophages, as key mediators of inflammation, have defined functionally important roles in mammalian tissue repair. Although recent evidence suggests that similar cells exist in zebrafish and also migrate to sites of injury in larvae, whether these cells are functionally important for wound healing or regeneration in adult zebrafish is unknown. To begin to address these questions, we first tracked neutrophils (*lyzC^+^*, *mpo^+^*) and macrophages (*mpeg1^+^*) in adult zebrafish following amputation of the tail fin, and detailed a migratory timecourse that revealed conserved elements of the inflammatory cell response with mammals. Next, we used transgenic zebrafish in which we could selectively ablate macrophages, which allowed us to investigate whether macrophages were required for tail fin regeneration. We identified stage-dependent functional roles of macrophages in mediating fin tissue outgrowth and bony ray patterning, in part through modulating levels of blastema proliferation. Moreover, we also sought to detail molecular regulators of inflammation in adult zebrafish and identified Wnt/β-catenin as a signaling pathway that regulates the injury microenvironment, inflammatory cell migration and macrophage phenotype. These results provide a cellular and molecular link between components of the inflammation response and regeneration in adult zebrafish.

## INTRODUCTION

In mammals, distinct cells of the inflammatory response play crucial roles in determining the level of repair of injured organs. Neutrophils contribute to the initial defense against foreign microbes and their ultimate removal (resolution) is essential for optimal tissue repair (Martin and Feng, 2009; Novoa and Figueras, 2012). Macrophages, comprising distinct subpopulations of M1 or M2 subtypes, secrete growth factors and cytokines that may attract keratinocytes and fibroblasts to trigger either tissue repair or scar formation (Leibovich and Ross, 1975; Serhan and Savill, 2005; Sica and Mantovani, 2012; Murray and Wynn, 2011). Neutrophils and macrophages can have pro- or anti-repair effects after injury, depending on the tissue and injury context (Dovi et al., 2003; Brancato and Albina, 2011; Marrazzo et al., 2011; Walters et al., 2009). Therefore, it is evident that modulating inflammation could be a useful therapeutic approach to augment tissue healing.

Mammals have a limited capacity for regeneration (Porrello et al., 2011; Seifert et al., 2012). In light of evidence that tissue regeneration is an evolutionarily conserved response to injury (Morrison et al., 2006), this has provided an incentive to identify useful models relevant to mammalian inflammation for the study of regeneration. Zebrafish have become a powerful vertebrate model for understanding the cellular and molecular mechanisms of regeneration (Goldsmith and Jobin, 2012) based on their regenerative ability, their simple but relevant anatomy, *in vivo* imaging capability and genetic advantages. The adult zebrafish tail (caudal) fin has become a model of choice for studying analogous appendage regeneration in mammals. The caudal fin is composed of bony rays, mesenchymal tissue, blood vessels and nerves, enclosed by epidermis and can fully regenerate all tissues after resection. Regeneration of the caudal fin after amputation (resection) entails three regenerative stages: (1) wound healing [0-1 days post amputation (dpa)]; (2) formation of the regeneration blastema (1-3 dpa), a mass of highly proliferative lineage-restricted mesenchymal progenitor cells; and (3) regenerative outgrowth and patterning of new tissue (>3 dpa) (Echeverri et al., 2001; Han et al., 2005; Kintner and Brockes, 1984; Stoick-Cooper et al., 2007a,b).

Several signaling pathways are known to control different aspects of the regenerative process. Of particular note is Wnt/β-catenin signaling, which is necessary and sufficient for caudal fin regeneration (Kawakami et al., 2006; Stoick-Cooper et al., 2007a,b). Given the crucial role of Wnt/β-catenin signaling in zebrafish fin regeneration, as well as evidence that this pathway regulates macrophage chemotaxis, recruitment and inflammatory diseases in several mammalian models (Newman and Hughes, 2012; Matzelle et al., 2012; Baker-LePain et al., 2011; Whyte et al., 2012), Wnt/β-catenin signaling is a candidate for linking inflammation and regeneration in zebrafish. However, it is still relatively unclear how this key pathway is activated and how Wnt/β-catenin signaling affects specific cells and stages of the regenerative process.

Importantly, zebrafish share many features with the mammalian immune system, including the existence of cells analogous to neutrophils, macrophages, dendritic cells and B and T cells (Renshaw and Trede, 2012). Zebrafish neutrophils rapidly accumulate at wounds in larvae through various injury cues and engulf small dead cell debris, much like their mammalian counterparts (Renshaw et al., 2006; Loynes et al., 2010; Mathias et al., 2007; Li et al., 2012; Yoo et al., 2011; Colucci-Guyon et al., 2011). Larval zebrafish macrophages appear at wound sites later than neutrophils, exhibit phagocytic behavior in response to bacterial infiltration and, as in mammals, may exist as different subsets of differing function (Herbomel et al., 1999; Lieschke et al., 2011; Redd et al., 2006; Mathias et al., 2009; Volkman et al., 2010). These larval studies indicate that these inflammatory cells may behave similarly after injury to their mammalian counterparts. A number of transgenic lines have been developed that express fluorescent reporters under the control of neutrophil [*myeloperoxidase* (*mpo*; *mpx* – ZFIN); *lysozyme C* (*lyzC*)] and macrophage-driven [*macrophage expressed 1* (*mpeg1*)] promoters in order to better characterize the injury response of these cells (Mathias et al., 2006, 2009; Ellett et al., 2011).

Nonetheless, the functional role of these cells in adult zebrafish tissue regeneration is still unclear. Intriguingly, inflammation may be a positive regulator of zebrafish neuronal regeneration in traumatic brain injury (Kyritsis et al., 2012), which is contrary to findings in mammals. Dissecting out the effect(s) of individual inflammatory components on regeneration is a more useful approach to understanding how inflammation may be involved in the regenerative process. Moreover, detailing the cellular inflammatory response after injury, its effect on zebrafish regeneration, and the molecular mechanisms involved is crucial in driving forward the study of vertebrate immunity in general.

The present study uses transgenic cell tracking and genetic ablation technology to identify the *in vivo* post-injury response of neutrophils and macrophages, as well as delineating functional roles of macrophages in zebrafish caudal fin regeneration. Our findings provide evidence for stage-dependent functional roles of macrophages in the regenerative process, shed light on possible signaling cues that modulate this response, and provide a context-specific functional link between inflammation and regeneration in adult zebrafish.

## RESULTS

### Neutrophils and macrophages are differentially recruited during fin regeneration

In order to characterize the cellular inflammatory response that occurs during adult caudal fin regeneration in zebrafish, we used transgenic fish to track the two most prominent types of inflammatory cells, namely neutrophils and macrophages. Neutrophils were visualized with Tg(*mpo*:GFP) and Tg(*lyzC*:dsRed) fish, in which cellular fluorescence is driven by the *mpo* and *lyzC* promoters, respectively (Mathias et al., 2006; Renshaw et al., 2006; Hall et al., 2007), and these largely label the same cells (supplementary material Fig. S1). Macrophages were visualized using Tg(*mpeg1*:mCherry) fish, with *mCherry* expression driven by the *mpeg1* promoter (Ellett et al., 2011). Recent studies have extensively characterized the specificity of these neutrophil and macrophage promoters (Ellett et al., 2011; Mathias et al., 2006, 2009).

To visualize these inflammatory cells throughout regeneration, caudal fins were amputated and live images were taken at various time points starting from 6 h post amputation (hpa) and continuing through 14 dpa. In addition to characterizing general inflammation throughout adult fin regeneration, we compared inflammatory responses in tissue undergoing differing rates of regeneration within the same fin in order to better understand how inflammation correlates with regeneration. To accomplish this, we used the inherent positional memory of amputated fins (Lee et al., 2005; Nachtrab et al., 2013) and performed both proximal (rapid growth) and distal (slow growth) resections within individual fish fins. During regeneration, undamaged cells retain or actively use information that may dictate morphological pattern, a phenomenon termed positional memory. Quantification of inflammatory cells was by total fluorescence intensity normalized to the injured area (see Materials and Methods).

**Fig. 1.**
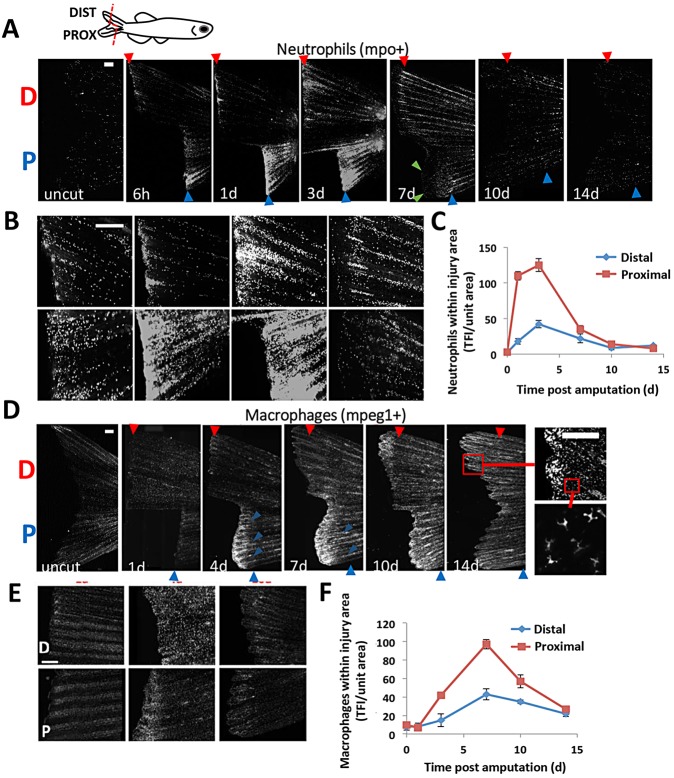
**Leukocyte recruitment in regenerating caudal fins follows distinct timelines and aligns with positional memory.** (A,B) Representative images detailing a regenerative timecourse of neutrophil accumulation in Tg(*mpo*:GFP) amputated fish, from uncut through 14 dpa. Fish received a dorsal proximal cut (indicated by ‘P’) and a ventral distal cut (‘D’). Fluorescent images were acquired and converted to grayscale for visualization. (C) Neutrophil density was quantified separately for the resected edge of both the proximal and distal cuts (*n*=9). Total fluorescence intensity of GFP-positive cells was normalized to the injured fin area and used as a correlation for cell number (see Materials and Methods). TFI, total fluorescence intensity. (D,E) Using the same strategy as above, macrophages were tracked in Tg(*mpeg1*:mCherry) fish during 14 days of regeneration. Boxes indicate regions magnified. (F) Quantification of macrophages near the amputation planes for proximal and distal cuts (*n*=10). Both neutrophils and macrophages accumulate in greater numbers in more proximal (faster regenerating) compared with distally cut tissue. Error bars indicate s.e.m. averages of each experiment. Scale bars: 200 µm.

Consistent with an early role in response after injury, neutrophil accumulation began at 6 hpa in adult Tg(*mpo*:GFP) fish (Fig. 1A-C). Peak accumulation was achieved by 3 dpa, with the number of localized neutrophils rapidly declining by 5 dpa. Pre-amputation levels of neutrophils were reached by 7 dpa and maintained through 14 dpa (Fig. 1A-C). Proximal amputations recruited over twice the number of neutrophils as distal amputations, but both injuries followed the same pattern of accumulation throughout regeneration. Similar to larval fins and most mammalian tissues, few neutrophils were resident in uninjured adult fin tissue. Neutrophil recruitment appeared to be driven by departure from the vasculature near the amputation plane, followed by migration to the injured area (supplementary material Movie 1). A similar accumulation pattern was seen in experiments with the alternative neutrophil tracking fish, Tg(*lyzC*:dsRed) (supplementary material Fig. S2).

Using the same strategy as above, we amputated caudal fins of the Tg(*mpeg1*:mCherry) fish to track macrophage behavior during regeneration. In contrast to neutrophils, macrophages were resident in greater density than neutrophils in uninjured fin tissue and showed little localized accumulation through 3 dpa (Fig. 1D,E). Macrophages began accumulating near the injured edge at 3-4 dpa, reached their peak numbers at ∼6-8 dpa and gradually decreased through 14 dpa (Fig. 1D-F). Again contrasting with neutrophils, macrophages appeared to accumulate primarily in newly regenerated tissue (Fig. 1D,E, 4-7 dpa, green arrows mark the proximal boundary of new fin tissue) and maintained elevated levels even at 14 dpa (Fig. 1F). Both neutrophils and macrophages accumulated more quickly and at greater densities in the more proximal (faster regenerating) resection compared with distally amputated tissue (Fig. 1C,F).

Although no published means exists to inhibit macrophage recruitment, we did investigate how reducing neutrophil recruitment after injury might affect fin regeneration. Incubation in diphenyliodonium chloride (DPI), a hydrogen peroxide inhibitor previously shown to inhibit neutrophil recruitment to injury (Deng et al., 2012; Yoo et al., 2011), reduced neutrophil accumulation to the injury site through 3 dpa, yet yielded no difference in the rate of fin regeneration compared with untreated fish (supplementary material Fig. S4).

In summary, both neutrophils and macrophages are present at the right time and location to be functionally involved fin regeneration, as we examine below.

### Genetic ablation of macrophages reveals a functional role during regeneration

To investigate the functional role of macrophages in fin regeneration we developed a transgenic fish Tg(*mpeg1*:NTR-eYFP) that utilizes an eYFP-tagged, human codon-optimized version of the *Escherichia coli* enzyme nitroreductase (NTR) downstream of the *mpeg1* promoter. NTR converts an exogenously delivered pro-drug metronidazole (MTZ) into a cytotoxic agent capable of killing the cell. NTR-MTZ ablation technology has been used in zebrafish to successfully ablate a variety of specific cells and tissues in both larval and adult zebrafish with negligible neighboring effects (Chen et al., 2011; Curado et al., 2007; Singh et al., 2012) (supplementary material Fig. S5A).

After 36 h of MTZ treatment, the numbers of cells showing *mpeg1*-driven fluorescence in Tg(*mpeg1*:NTR-eYFP) fish were, upon visual inspection, dramatically reduced throughout most discernible tissues including the eye, pectoral fin and caudal fin. We quantified the reduction of macrophages in the caudal fin by flow cytometry, and consistently obtained ∼80-90% reduction of eYFP^+^ cells in MTZ-treated Tg(*mpeg1*:NTR-eYFP) fish compared with untreated fish (supplementary material Fig. S5B,C and Fig. S6). eYFP^+^ cells were morphologically identical to mCherry^+^ cells in Tg(*mpeg1*:mCherry) fish, and the migrational timeline of eYFP^+^ cells during fin regeneration was also identical to that of mCherry^+^ cells, indicating that the Tg(*mpeg1*:NTR-eYFP) line is macrophage specific (supplementary material Fig. S5A,D). We did not observe any unusual behavior, including aberrant swimming or eating behavior, in these animals.

Macrophage recovery was initiated by washing out the MTZ with regular fish water. Washout resulted in a return to normal macrophage levels, which is indicative of a constant renewal model of macrophage replacement (supplementary material Fig. S6B and Fig. S9). Continuous drug treatment daily for up to 14 dpa resulted in >80% ablation during and at the end of the timecourse (supplementary material Fig. S9). We tested for deleterious unintended effects of MTZ drug treatment by first quantifying the number of caspase 3^+^ (apoptotic) cells in the caudal fin in wild-type adult fish before and after continuous MTZ treatment and no difference was found (supplementary material Fig. S7A). Moreover, no morphological differences in new fin tissue after caudal fin amputation were observed after treatment with MTZ in wild-type fish (data not shown). Finally, inflammation was not affected by MTZ treatment in wild-type fish that had undergone fin amputation (supplementary material Fig. S7B,C and Fig. S8). Thus, this macrophage ablation model exhibits minimal off-target effects.

**Fig. 2.**
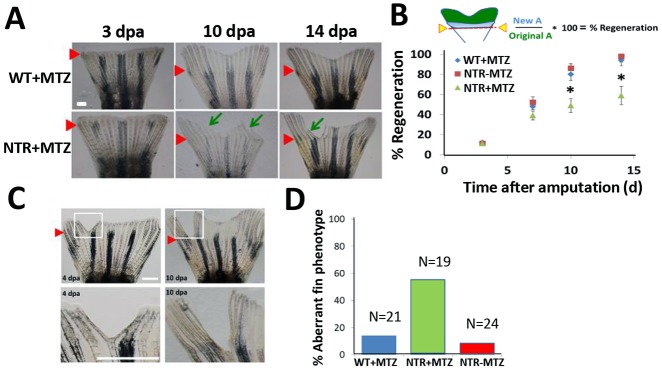
**Macrophages modulate caudal fin regeneration rate and phenotype.** (A) Macrophages were continuously ablated after fin resection (up to 14 dpa) using the macrophage ablation fish line Tg(*mpeg1*:NTR-eYFP). Fin images are representative of macrophage-ablated (NTR+MTZ) and control (WT+MTZ) fish in at least three independent experiments. Green arrows point to areas of unusually reduced tissue growth and formation; red arrowheads indicate the original fin cut line. (B) Quantification of regenerated tissue as a percentage of original fin area for NTR+MTZ (*n*=11), WT+MTZ (*n*=18) and control fish (NTR−MTZ, *n*=14). Full regeneration to the original fin area is considered 100% regeneration. Data are compiled and averaged over three separate experiments using identical conditions. 10 dpa, *P=0.0124; 14 dpa, *P=0.0262; two-tailed *t*-test. Error bars indicate s.e.m. averages of each experiment. (C) Representative images at 4 dpa and 10 dpa of MTZ-treated Tg(*mpeg1*:NTR-eYFP) caudal fins displaying aberrant tissue phenotypes. (D) Summary of percentage of fish qualitatively assessed for aberrant phenotypes at 14 dpa. Scale bars: 300 µm.

To examine the regenerative capacity of the tail fin after substantial macrophage loss, we amputated caudal fins from wild-type and Tg(*mpeg1*:NTR-eYFP) fish and continuously treated both with MTZ for 14 dpa. In transgenic fish in which macrophages were ablated (NTR+MTZ), the extent of new fin tissue growth was decreased compared with wild-type fish given drug daily (WT+MTZ) (Fig. 2A,B). Tg(*mpeg1*:NTR-eYFP) fish that were fin amputated but did not receive MTZ treatment had regeneration rates similar to those of wild type (Fig. 2B). Moreover, new fin tissue growth was often non-homogeneous in NTR+MTZ fish. These fish often displayed scattered, distinct areas of aberrant tissue growth along the fin (Fig. 2A, green arrows mark areas of comparatively reduced growth), which can occur normally, at a rate significantly higher (56%) than in WT+MTZ (13.4%) or NTR−MTZ (7.8%) (Fig. 2D). We conducted a similar experiment using a larval fold fin amputation model and observed a slight decrease in new tissue at 5 dpa (supplementary material Fig. S10), which is suggestive of at least a partially conserved role in regeneration from larvae to adults.

**Fig. 3.**
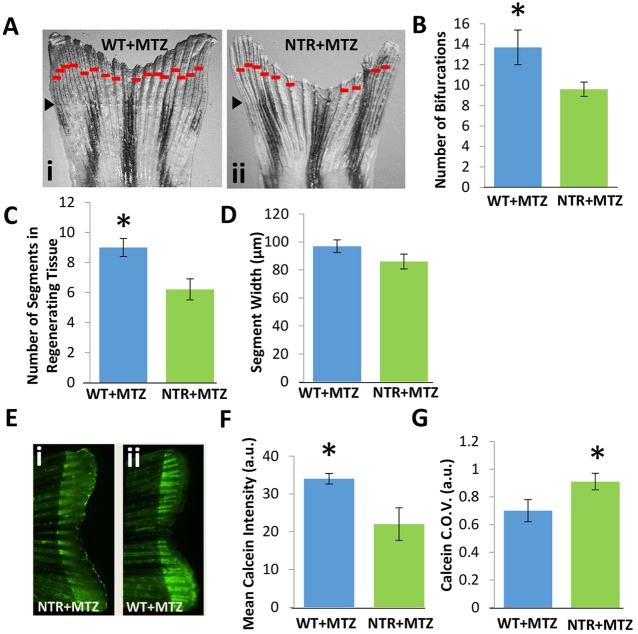
**Macrophages modulate bony ray patterning and formation during tissue outgrowth.** Macrophages were continuously ablated up to 10 dpa. (A) Representative fin images of NTR+MTZ (ii) versus control (i) for at least two independent experiments. Red bars indicate bifurcation points on each ray. Black arrowheads indicate the original fin cut line. (B) Total bifurcations in regenerated tissue are decreased in NTR+MTZ fish compared with wild-type fish. *P=0.030 (two-tailed *t*-test, error bars indicate s.e. m.). (C) The average number of total segments in each regenerated bony ray is decreased in NTR+MTZ fish compared with WT+MTZ fish. *P=0.040 (two-tailed *t*-test, error bars indicate s.e.m.). (D) Average segment width for NTR+MTZ and control fins. No significant differences were observed. (E) Fluorescent images of calcein staining in (ii) WT+MTZ and (i) NTR+MTZ fish. Note the less intense and more scattered staining in NTR+MTZ fins compared with WT+MTZ fins. (F) Mean calcein intensity is decreased in NTR+MTZ fish compared with WT+MTZ fish. *P=0.044 (two-tailed *t*-test, error bars indicate s.e.m.). (G) Coefficient of variation (C.O.V.; a measure of dispersion) for calcein intensity is significantly increased in NTR+MTZ fish compared with wild-type fish. *P=0.047 (two-tailed *t*-test, three separate experiments, error bars indicate s.e.m.).

Since each bony ray can regenerate independently of others, we also examined how macrophage depletion alters individual bone ray length segment morphology and ray branching. Quantitative image analysis at 10 dpa revealed that NTR+MTZ fish exhibited a significant reduction in the average number of segments in the regenerated ray (*P*<0.04, Fig. 3A,C), although bone segment width was not significantly altered (Fig. 3D). Bone ray branching (as measured by the number of bifurcations) was also altered in NTR+MTZ fish (*P*<0.03, Fig. 3B), and joint specification (bifurcation position) was unchanged. These latter data specify direct measures of bone patterning, since osteoblast activity can only partially affect these measures (Knopf et al., 2011). We further investigated bone quality, via mineralization formation, using *in vivo* calcein labeling to examine actively mineralizing surfaces in newly regenerated bone segments. Qualitatively, NTR+MTZ fish exhibited greater inter-ray heterogeneity and weaker calcein labeling than WT+MTZ fish in the regenerated tissue (Fig. 3E). We quantified calcein intensities in individual bone segments. Quantification of the coefficient of variation of intensity (Fig. 3G), which is a measure of dispersion, supported the qualitative assessment that NTR+MTZ induced a greater heterogeneity and reduced intensity of labeling (Fig. 3F). Taken together, these data indicate that macrophage depletion impairs bone ray patterning and the quality of bone formation.

**Fig. 4.**
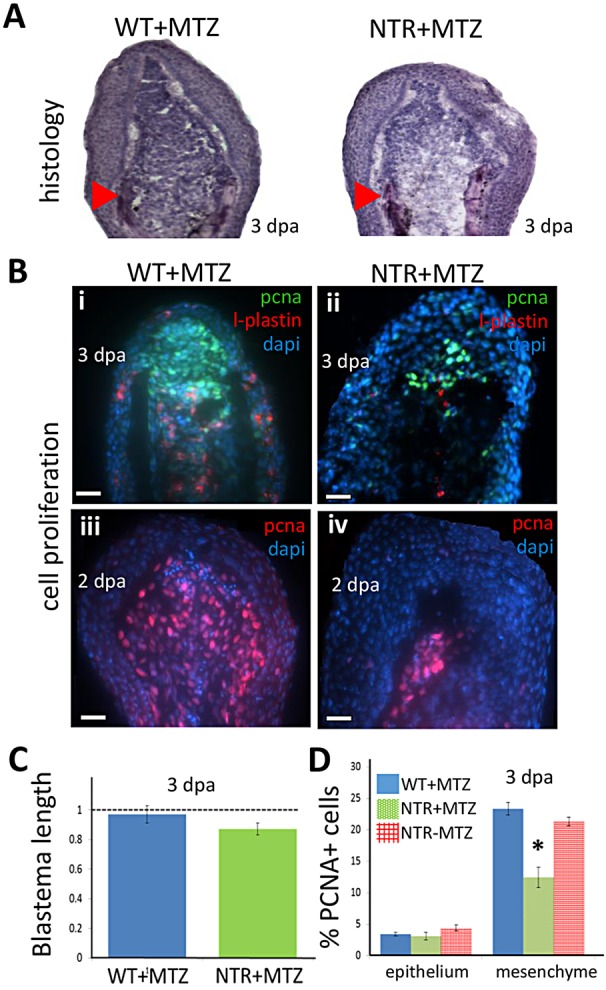
**Macrophages modulate the proliferative capacity of the regeneration blastema.** (A) Hematoxylin-stained sections of tail fin regenerates (blastemal region) at 3 dpa. Macrophage-depleted fins (right) display slightly reduced numbers of deep mesenchymal cells of the blastema. Arrowheads indicate the plane of amputation. (B) Blastemal and macrophage proliferation assessed by staining 2 (iii,iv) or 3 (i,ii) dpa regenerates for PCNA (i-iv) or L-plastin (i,ii), a marker for leukocytes (mostly macrophages), and with DAPI. Scale bars: 20 µm. (C) Quantification of the length of the blastema in macrophage-depleted (NTR+MTZ; *n*=7) and wild-type (*n*=6) fins at 3 dpa. Macrophage-depleted fins displayed slightly decreased blastemal size compared with wild-type fins. (D) Cell proliferation (PCNA^+^ cells) quantified in the blastema is reduced in NTR+MTZ compared with wild-type controls. PCNA^+^ cell number was averaged among all sections spanning the entire fin width, and normalized to DAPI counts in the image. WT+MTZ, *n*=10; NTR−MTZ, *n*=8; NTR+MTZ, *n*=9. *P=0.0425 (two-tailed *t*-test, error bars indicate s.e.m.).

We next investigated how macrophages might affect key regenerative processes. We concentrated on possible effects of macrophages on blastema phenotype and function, particularly proliferative capacity. We amputated caudal fins from wild-type and Tg(*mpeg1*:NTR-eYFP) fish and continuously treated both with MTZ for 3 dpa throughout blastema formation. We observed that a loss of macrophages did not significantly affect gross blastema morphology and size (Fig. 4A,C), but did result in a significant decrease in actively proliferating cells, particularly in the mesenchymal region (Fig. 4B,D). We also assayed gene expression levels from blastema regions of macrophage-depleted fins and detected reduced levels of regeneration-associated genes, along with various injury-response genes, particularly at 4 dpa (supplementary material Fig. S11). To investigate whether macrophages affect other components of inflammation, we continuously depleted macrophages before and after injury in Tg(*lyzC*:dsRed) and Tg(*mpeg1*:NTR-eYFP; *lyzC*:dsRed) fish and did not observe significantly altered neutrophil accumulation or resolution (supplementary material Fig. S7B and Fig. S8). Taken together, these data indicate that macrophages affect the rate of caudal fin regeneration possibly through impacting the proliferative capacity of the blastema.

### Macrophages exhibit stage-dependent effects on fin regeneration

**Fig. 5.**
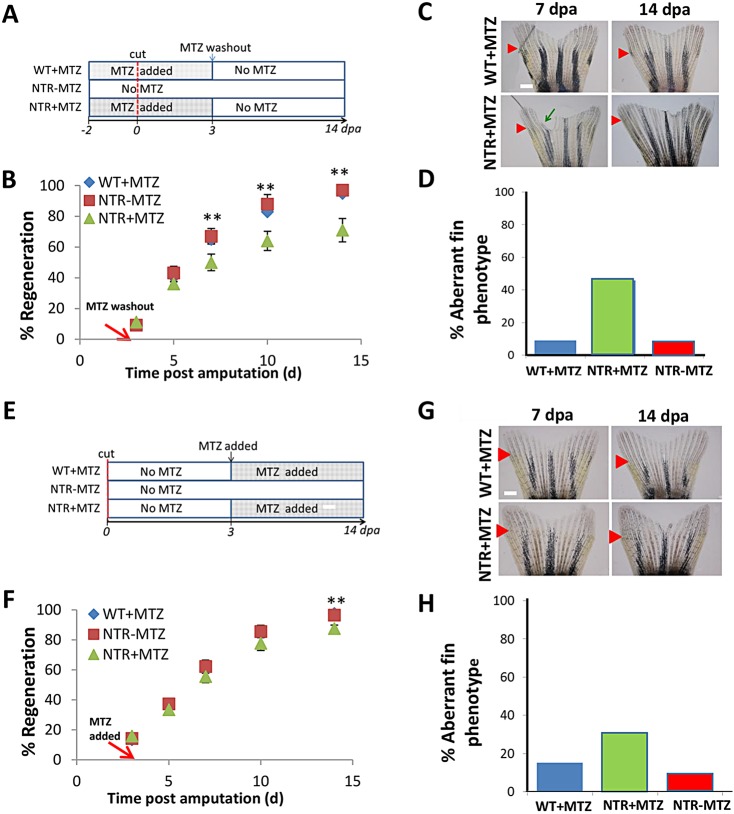
**Macrophages exhibit stage-dependent effects on fin regeneration.** (A) Experimental scheme. Macrophages were ablated after fin resection through 3 dpa, then allowed to repopulate normally via MTZ washout. (B) Representative fin images at 7 and 14 dpa, which is 4 and 11 days after macrophage repopulation initiation, respectively. Green arrow indicates irregular fin phenotype, as dictated by non-homogenous growth areas; red arrows indicate original resection plane. (C) Macrophage reduction through 3 dpa largely recapitulated the reduction in regenerative outgrowth seen with 14 days ablation. Rate of tissue regeneration was reduced in NTR+MTZ (*n*=11) fish compared with WT+MTZ (*n*=7) and NTR-MTZ (*n*=10) fish. Data are averaged over two separate experiments using identical conditions. 7 dpa, **P=0.0455; 10 dpa, **P=0.0278; 14 dpa, **P=0.0220; two-tailed *t*-test. (D) Quantification of percentage of fish displaying any aberrant phenotype at 14 dpa. Total quantification is cumulative from two separate experiments. (E) Experimental scheme. Macrophages were ablated beginning at 3 dpa through 14 dpa. (F) Representative images at 7 and 14 dpa, which is 4 and 11 days after the ablation of macrophages had begun, respectively. (G) Delayed macrophage reduction did not significantly reduce the rate of regeneration. Data are averaged over two separate experiments using the same conditions. (H) Quantification of the percentage of fish displaying any aberrant phenotype at 14 dpa. Data are cumulative from two separate experiments. Error bars indicate s.e.m. Scale bars: 300 µm.

We took advantage of the cell recovery utility of this model to explore when macrophages are required for complete fin regeneration. We ablated macrophages at two distinct time frames during fin regeneration. To test their requirement during blastema formation and wound healing, we ablated macrophages beginning 2 days before amputation through 3 dpa, followed by washout until 14 dpa (Fig. 5A), during which new macrophages were produced and migrated to the fin (supplementary material Fig. S6B, Fig. S9). When macrophages were ablated through blastema formation (−2 to 3 dpa), regeneration was inhibited to a similar extent as ablating macrophages for the entire 14-day post-resection period (Fig. 5A-C). Moreover, aberrant fin phenotypes persisted in macrophage-depleted fish (Fig. 5D). To test macrophage requirement during tissue outgrowth, we ablated from 3 dpa through 14 dpa (Fig. 5E); the regeneration rate was not significantly affected (Fig. 5F,G). The occurrence of the aberrant phenotype was still elevated in macrophage-depleted fish (33%, NTR+MTZ) over controls (16%, WT+MTZ; 9%, NTR−MTZ). Thus, there is a functional requirement for macrophages during the wound healing and blastema formation stage that directly affects subsequent tissue growth, whereas during the tissue outgrowth stage macrophages mainly modulate only tissue patterning.

### Wnt/β-catenin signaling modulates the recruitment and resolution of inflammatory cells

**Fig. 6.**
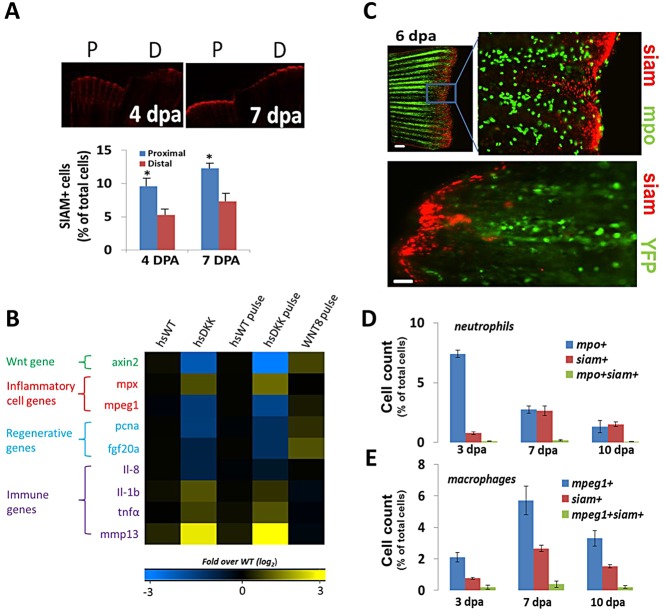
**Wnt/β-catenin signaling by non-leukocytes affects the injury environment in regenerating fins.** (A) Representative images detailing cells undergoing Wnt/β-catenin signaling (*siam*^+^, red) for proximal and distal fin resections in Tg(*TCFsiam*:mCherry) fish. *Siam^+^* cell number is increased in proximal cuts. 4 dpa, *P=0.0329; 7 dpa, *P=0.0296 (two-tailed *t*-test, error bars indicate s.e.m.). (B) Gene expression levels (4 dpa) of pooled blastemal fin tissue (*n*>5) as assessed by qRT-PCR for wild-type and for the Tg(*hsDKK1*:GFP) loss-of-function and Wnt8a (*hsWnt8a*:GFP) gain-of-function Wnt/β-catenin signaling fish lines. Levels were normalized to fold over non-heat shock control. Data were averaged over two separate experiments. One group included daily heat shock following amputation; the other group included a single heat shock pulse at 84 hpa with tissue extraction 12 h later at 4 dpa. *mpx* is *mpo*. (C) Representative images of distal resections from Tg(*mpo*:GFP; *TCFsia*m:mCherry) fish and Tg(*mpeg1*:NTR-YFP; *TCFsiam*:mCherry) fish at 6 dpa. Little colocalization is evident between neutrophils (*mpo*^+^) and *siam*^+^ cells. Scale bar: 40 µm; 100 µm in bottom panel. (D) Quantification of flow cytometry sorted cells from pooled resected fins (*n*=8) from Tg(*mpo*:GFP; *TCFsiam*:mCherry) fish indicating the presence of few *mpo*^+^ *siam*^+^ cells. (E) Quantification of flow cytometry sorted cells from pooled resected fins (*n*=7) from Tg(*mpeg1*:*NTR*-eYFP; *TCFsiam*:mCherry) fish indicating the presence of few *mpeg1^+^ siam^+^* cells. (D,E) Error bars indicate s.e.m. of the average of three experiments.

Since Wnt/β-catenin signaling is required for blastema formation and regenerative outgrowth in zebrafish caudal fins (Ito et al., 2007; Kawakami et al., 2006; Poss et al., 2000; Stoick-Cooper et al., 2007a,b), but also modulates inflammatory processes including scar formation, fibrosis, wound healing and tissue remodeling in mammals (French et al., 2004; Ren et al., 2013; Koch et al., 2011), we investigated whether there might be a role for Wnt/β-catenin signaling in regulating inflammation during fin regeneration. Using a transcriptional reporter line of Wnt/β-catenin signaling, Tg(*7xTCF-Xla.Siam:nlsmCherry*)^ia5^ [designated Tg(*TCFsiam*:mCherry); Moro et al., 2012], which expresses nuclear-localized mCherry driven by a multimerized TCF response element and minimal *siamois* promoter, we tracked cells undergoing active Wnt/β-catenin signaling. We discovered that a greater density of these cells resides in proximal (faster regenerating) than distal (slower regenerating) resections, similar to the trend of neutrophil and macrophage densities (Fig. 1 and Fig. 6A). In order to directly assess the effect of Wnt/β-catenin signaling on the injury response, we assessed gene expression levels in blastema fin tissue in a transgenic line expressing heat shock-inducible Dickkopf (*hsDKK1*:GFP), a secreted inhibitor of Wnt/β-catenin signaling, and Wnt8a (*hsWnt8a*:GFP). Genes characteristic of the early injury response (*tnfa*, *il1b*, *mmp13*) were upregulated in DKK1-overexpressing fish over wild-type controls, either during continuous Wnt inhibition or after a 12 h pulse (Fig. 6B). Levels were unchanged when Wnt8a was overexpressed for 12 h (Fig. 6B), implying that a Wnt/β-catenin signaling threshold might modulate the injury microenvironment.

To determine if Wnt/β-catenin signaling acts directly on inflammatory cells in this context, we crossed the Tg(*TCFsiam*:mCherry) Wnt reporter fish line with the neutrophil-tracking Tg(*mpo*:GFP) fish line and separately with the Tg(*mpeg1*:\TR-eYFP) macrophage ablation line. Inflammatory cells accumulated near *siam^+^* cells distally, but did not appear to express *mCherry* (Fig. 6C). Using flow cytometry on pooled, dissociated fins, we found that fewer than 1% of neutrophils and 3% of macrophages exhibited activated Wnt reporter fluorescence at 3, 7 or 10 dpa, indicating that the substantial majority of inflammatory cells do not display elevated Wnt/β-catenin signaling (Fig. 6D,E). Hence, the effects of Wnt signaling on cytokine expression are mediated through a non-leukocyte, as yet unidentified, cell population.

**Fig. 7.**
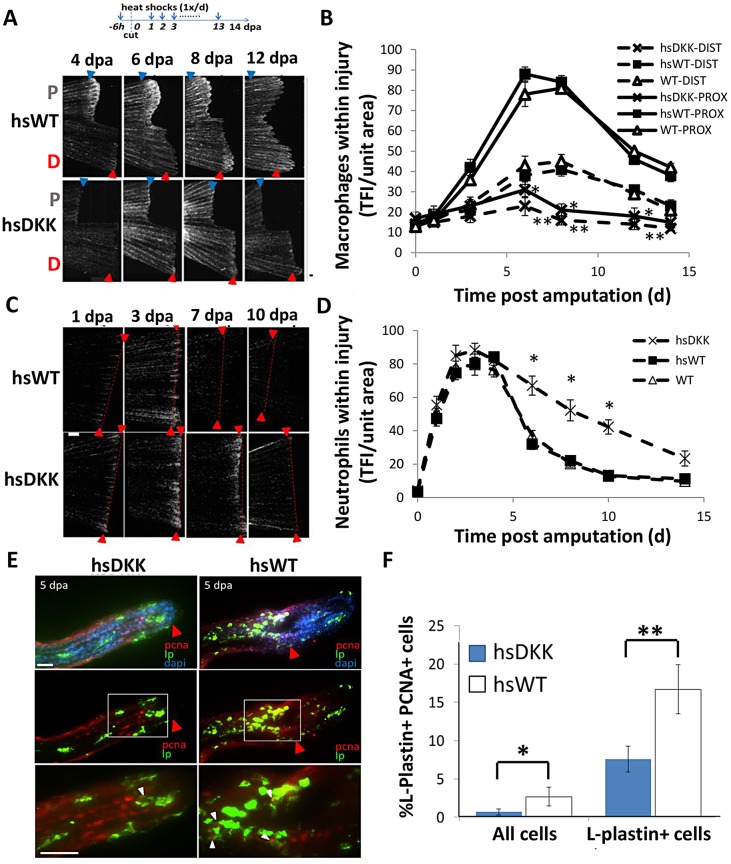
**Wnt/β-catenin signaling regulates leukocyte response to injury.** (A) The loss-of-function Wnt/β-catenin signaling line Tg(*hsDKK1*:GFP) crossed to the Tg(*mpeg1*:mCherry) line was used to track macrophages after Wnt modulation. Resected wild-type or loss-of-function Wnt/β-catenin signaling (hsDKK) fins received a proximal cut and a distal cut. Representative images are shown of macrophage accumulation through 12 dpa. Fluorescent images were acquired and converted to grayscale for ease of visualization. (B) Macrophage accumulation was reduced in DKK1-overexpressing fins at every time point from 3 dpa until 14 dpa and no significant difference in macrophage number was observed between proximal and distal resections. Data are representative of at least three independent experiments with at least six to eight fish per time point. HsDKK-PROX versus hsWT-PROX, WT-PROX: 6 dpa, *P=0.0083; 8 dpa, *P=0.0072; 12 dpa, *P*=0.0175. HsDKK-DIST versus WT-DIST, WT-DIST; 6 dpa, **P=0.0140; 8 dpa, **P=0.0195; 12 dpa, **P=0.0361; two-tailed *t*-test. (C) Tg(*hsDKK1*:GFP) was crossed to a neutrophil promoter-driven Tg(*lyzC*:dsRed) line in order to visualize neutrophil accumulation following Wnt inhibition. Representative images indicate that neutrophil accumulation remains elevated longer in DKK1-overexpressing fins compared with wild-type controls. (D) Neutrophil accumulation was higher in DKK1-overexpressing fins compared with wild-type controls after 5 dpa. Data are representative of three independent experiments with at least six to eight fish per time point/condition. hsDKK1 versus hsWT, WT: 6 dpa, *P=0.0075; 8 dpa, *P=0.0112; 10 dpa, *P=0.0105; two-tailed *t*-test. (E) Proliferation of wild-type and DKK1-overexpressing regenerates at 5 dpa as assessed by anti-PCNA (red), anti-L-plastin (green) and DAPI (blue) staining. Red arrowheads indicate original cut site; white arrowheads indicate double-stained (PCNA^+^ LP^+^) cells. The boxed regions are magnified beneath. (F) Proliferating macrophages as a percentage of total cells and total macrophages (LP^+^ cells). Numbers were averaged over at least seven sections of each sample. Data are representative of three independent experiments (*n*>5). hsDKK1 versus hsWT: *P=0.0475; **P=0.0349 (two-tailed *t*-test, error bars indicate s.e.m.). Scale bars: 200 µm in A; 300 µm in C; 20 µm in E.

In order to assess the effect of Wnt/β-catenin signaling on inflammatory events, we crossed a transgenic line for heat shock-inducible Dickkopf (*hsDKK1*:GFP) with the Tg(*lyzC*:dsRed) neutrophil-tracking or Tg(*mpeg1*:mCherry) macrophage-tracking lines. Macrophage accumulation within the injured area was almost completely inhibited in Tg(*hsDKK1*:GFP) fish compared with wild-type fish (Fig. 7A,B). Moreover, unlike wild-type fish, in *hsDKK1*:GFP fish there was no significant statistical difference between proximal and distal resections in macrophage accumulation at any time period. The heat shock protocol by itself did not perturb inflammatory cell migration (Fig. 7B,D). Inhibition of Wnt/β-catenin signaling delayed neutrophil resolution and prolonged neutrophil number in the injury area compared with wild-type fish, taking twice as long (12 dpa) in DKK1-overexpressing fins to reach the level of neutrophils observed at 6 dpa for wild-type fins in adults (Fig. 7C,D). No cell accumulation differences were observed in gain-of-function Wnt8a fish compared with wild-type controls. To disassociate initial regenerative events from leukocyte migration later in the process, Wnt inhibition was delayed, beginning after tissue outgrowth initiation (at 3 and 5 dpa). Delayed Wnt inhibition again decreased macrophage accumulation near the site of injury (supplementary material Fig. S14). Furthermore, Wnt inhibition decreased the density of proliferating macrophages (5 dpa) in the regenerating area (Fig. 7E,F; supplementary material Fig. S12). Subsequent gene profiling of macrophages sorted from tissue subjected to a 12 h pulse of DKK1 resulted in gene expression profiles of known inflammation-associated cytokines [*il8* (*cxcl8*), *il10*, *il12*] that differed from wild-type control profiles (supplementary material Fig. S13).

Taken together, these data suggest that Wnt/β-catenin signaling might be necessary for normal progression of the injury response during regeneration. Moreover, this pathway may exert its effects mechanistically through modulating macrophage activity and phenotype at various time points.

## DISCUSSION

Although wound healing has been extensively studied in mammals, we have a limited understanding of the injury-induced cellular response in a regenerative context. In this study, we utilized a combination of cell tracking and genetic cell ablation approaches to detail the course and role of cellular components of inflammation in zebrafish fin regeneration. Our data suggest that the relative time frame of inflammatory cell movement to and from sites of injury is similar for adult zebrafish and mammals, where neutrophils are attracted to the wound first through ‘homing’ from the circulation, followed by circulation-based or resident macrophages (Sadik et al., 2011; Yoo and Huttenlocher, 2011; Li et al., 2012). Cell tracking data indicate that activated neutrophils are circulation derived, whereas most macrophages are resident in the fin, in contrast to both larval zebrafish and mammalian appendages. Macrophage accumulation mainly occurred after the blastema formation stage, suggesting that zebrafish macrophages respond to events well after the wound healing phase of fin regeneration. Therefore, we describe a fast-moving and fast-responding neutrophil population and a correspondingly slow-moving resident macrophage population in adult zebrafish.

We present evidence that macrophages may have differential stage-dependent effects on the extent of tail fin regeneration. Although mammalian macrophages serve unique, specific functions at distinct phases during tissue repair (Liu et al., 1999; Lucas et al., 2010), zebrafish macrophages seem to function differently at analogous stages after wounding. Whereas in mice macrophage depletion during tissue outgrowth can result in severe hemorrhage in the wound (Mirza et al., 2009), ablation during tissue outgrowth in zebrafish only affects fin patterning, not growth. Moreover, although macrophage depletion has not been found to negatively affect wound closure rates and endothelial repair in mammals (Dovi et al., 2003; Martin and Feng, 2009; Evans et al., 2013), macrophage depletion reduced tissue growth in adult zebrafish. We also found no evidence that zebrafish macrophages modulate neutrophil recruitment or resolution, whereas macrophages have been found to modulate these cellular responses in mouse limb wounds (Cailhier et al., 2006). These data provide further justification for the view that macrophages have different roles after appendage injury in mammals versus adult zebrafish.

This study supports the existence of either (1) a single macrophage population that has different roles in the regenerative course over time, or (2) multiple, functionally distinct macrophage populations, similar to in mammals. It is also possible that other myeloid-like cells might migrate from non-fin sites over the course of injury, although rapid macrophage movement was not observed either in vasculature or interstitial tissue. Macrophages mainly exerted effects on tissue growth during the initial regenerative stages, but aberrant phenotypes, including impaired bony ray patterning and bone formation, were still observed when depletion occurred after the tissue outgrowth phase (>3 dpa). These data advocate a model whereby spatially close resident macrophages modulate events initially, but during later regenerative stages either newly proliferated macrophages or slowly migrating macrophages affect the regenerative response in a different manner than the early macrophage population. Cataloguing the composition of this population over the injury timecourse using single-cell lineage tracing or Brainbow technology would be useful to delineate the level of macrophage heterogeneity.

In contrast to recent evidence that neutrophil deficiency (neutropenia) increases the regeneration rate in larval fins (Li et al., 2012), our creation of a neutropenic environment in adult zebrafish did not affect the fin regeneration rate. Moreover, it is unlikely that neutrophils have an inhibitory effect on regeneration because neutrophils accumulated in markedly greater numbers in faster regenerating tissue throughout the regenerative process. Since neutrophils may either promote or inhibit wound healing and tissue repair in mice depending on the tissue and injury context (Dovi et al., 2003; Harty et al., 2010; Marrazzo et al., 2011; Rieger et al., 2012), neutrophil function in zebrafish might be highly injury- and time-dependent. Given the proven utility of the genetic macrophage ablation model in this study, the creation of a similar *mpo-* and/or *lyzC*-driven ablation fish would more conclusively clarify the supportive or reductive role of neutrophils in various regenerative contexts.

We further establish that Wnt/β-catenin signaling partially modulates the time frame and degree of leukocyte response in tail fin regeneration. Wnt/β-catenin signaling inhibition ‘arrested’ the cell and cytokine environment at a stage similar to the early injury environment. Importantly, this effect was still observed when Wnt/β-catenin signaling was impaired after the initial regenerative events had begun, supporting a more direct role of Wnt signaling in determining macrophage movement. Active Wnt signaling might mitigate early stage inflammation and act as a molecular switch to proceed to later stages of the immune response (neutrophil resolution/macrophage enrichment). This idea shares similarities with the situation in mammals, in which timely neutrophil removal (resolution) after injury is essential to the termination of inflammation – delayed apoptosis or impaired clearance of neutrophils can aggravate and prolong tissue injury (Sadik et al., 2011). The idea that Wnt/β-catenin signaling may restrict several aspects of inflammation is supported in several mammalian models of disease and injury. For example, high Dkk1 activity is associated with pro-inflammatory bone loss in mouse myelomas (Tian et al., 2003), and inhibition of Dkk1 activity in a mouse model of rheumatoid arthritis results in greater bone formation (Diarra et al., 2007). The role of Wnt/β-catenin signaling in modulating the injury response might indeed be similarly context-specific in zebrafish; further study in other anatomical injury models would be beneficial in this context.

The cellular basis of the effects of Wnt signaling on inflammation is unclear, in part because cells responding to Wnt ligands had remained unidentified until very recently (Wehner et al., 2014); it was determined that a population of actinotrichia-forming cells and osteoblast progenitors undergo Wnt signaling during blastemal specification, regulating epidermal patterning and osteoblast differentiation indirectly through secretion of factors. Given that Wnt/β-catenin signaling inhibition eliminated the differential positional memory aspect of macrophage recruitment, and that delayed inhibition reduced longer term migration, it is likely that Wnt/β-catenin signaling also indirectly affects macrophage phenotype and activity through a similar regulation of secretion factors. Additionally, the similar accumulation patterns of Wnt-responsive cells (*siam^+^*) and neutrophils/macrophages suggests that both inflammatory and Wnt signaling cells might respond to the same injury signals. This idea is further supported by the fact that amputating more proximally also involves the damage of a greater volume of tissue and, therefore, may result in more robust levels of paracrine ‘injury signals’, including H_2_O_2_, redox and the Src family kinase Lyn, all previously identified in zebrafish (Pase et al., 2012; Yoo et al., 2012; Niethammer et al., 2009). Wnt-responding cells in mammals have recently been linked to modulating angiogenic factors, which can in turn affect the injury response (Kitajewski, 2003); examining whether Wnt inhibition and macrophage depletion regulate angiogenesis might shed more light on their mechanistic effects on inflammation and regeneration. Identifying which Wnt-modulated signals directly affect macrophage proliferation, cytokine release and migration would assist in further developing this mechanistic insight into how Wnt/β-catenin signaling modulates inflammation and regeneration.

Our findings detail the cellular events in the normal injury response during zebrafish epimorphic regeneration. We reveal that macrophages regulate aspects of appendage regeneration in adult zebrafish. We also provide evidence that Wnt/β-catenin signaling may in turn modulate cellular and biochemical inflammatory events during the regenerative process. Our findings, coupled with recent research detailing pro-repair roles of inflammatory cells in zebrafish brain regeneration, advocate some degree of anatomical conservation of the role of injury components in regenerative process in zebrafish. Finally, macrophages may indeed form part of a cellular bridge between robustly regenerative organisms such as zebrafish and the less regenerative mammals that could potentially be manipulated for mammalian regenerative therapies.

## MATERIALS AND METHODS

### Transgenic lines

The Tg(*mpeg1*:NTR-EYFP)^w202^ line was created using the Tol2 transposon system (Urasaki et al., 2006). Targeted cell ablation mediated by bacterial nitroreductase (NTR) was described previously (Curado et al., 2007). A DNA fragment containing EYFP-NTR was subcloned into a Tol2 vector that contained the zebrafish *mepg1* promoter (Ellett et al., 2011). The Tol2 construct and transposase RNA were microinjected into 1- to 4-cell stage embryos and the transgenic line was isolated by the specific expression of YFP in macrophages in the next generation. Tg(*hsDKK1*:GFP;*mpeg1*:mCherry), Tg(*hsWnt8a*:GFP;*mpeg1*:mCherry), Tg(*7xTCF-Xla.Siam*:nlsmCherry;*mpo*:GFP)^ia5^ (Moro et al., 2012), Tg(*lyzC*:dsRed;*mpo*:GFP) and Tg(*mpeg1*:NTR-EYFP;*7xTCF-Xla.Siam*:nlsmCherry) fish were made by crossing individual transgenic homozygotes with the corresponding transgenic complement.

### Adult zebrafish fin amputation surgeries

Zebrafish of ∼6-12 months of age were used for all studies. Fin amputation surgeries were performed as previously described (Stoick-Cooper et al., 2007a,b). Two amputation cut schemes were employed: (1) a single cut was made traversing the entire dorsoventral length of the caudal fin in each fish; or (2) two separate cuts were made on each fish, one closer to the body of the fish (‘proximal’, ventral) and one further away from the body (‘distal’, dorsal) (Lee et al., 2005).

### Live image analysis

The injured adult zebrafish were anesthetized as previously described with Tricaine (Stoick-Cooper et al., 2007a,b), placed on their side and imaged under a Nikon TiE inverted widefield fluorescence high-resolution microscope. Full fin images were assembled from 30-50 stitched images (20×) encompassing the entire fin, with the fish under constant anesthetization. Live fin images were taken for each fish periodically post amputation.

### Analysis of cell density in the injured area of amputated fins

To ascertain the timecourse of cell recruitment to the fin injury area, a measure of cell density near the resected fin edge was utilized. An ‘injured area’ was defined as the area spanning two set dimensions: one dimension being the distal-ventral boundary of the fin; the other dimension being defined as from perpendicular to the distal-ventral axis, one-quarter of the fin length proximal to the original amputation plane. Using Image-Pro software (Media Cybernetics), the total fluorescence intensity (TFI) from promoter-driven fluorescent cells in the injury area from fin images at each time point was quantified. The TFI was normalized to the pixel area of the injured area for that fin to obtain a measure of cell density in the injured area. This analysis was used based on the assumption that the fluorescence intensity of each labeled cell was similar on average in each fish as verified by flow cytometry.

### Fin regeneration measurements

Total regeneration was gauged by a percent regeneration metric. Briefly, this measurement required phase-contrast full-fin images be taken before amputation and at each time point after amputation. The full area (in pixels) of the caudal fin, from the proximal end of the fin rays to the distal fin edge/cut, was quantified from the pre-amputation images for each fish using ImageJ (NIH). The new tissue area, from the new distal fin edge to the amputation plane, was also quantified. Percent regeneration for each fin at each time point was defined as: % regeneration=100×(new tissue area/original fin area amputated).

### Macrophage ablation

For all macrophage ablation experiments, Tg(*mpeg1*:NTR-eYFP) fish were housed in static tanks of fish water (five fish/liter) supplemented with or without 2.5 mM metronidazole (MTZ) for the duration of the experiment. During ablation experiments, fish were kept on a 12 h light/12 h dark cycle, since MTZ is sensitive to long exposure to light. Water was changed daily and fresh MTZ was added daily. Two control groups were used: NTR transgenic fish housed in fish water without MTZ, and wild-type fish housed in fish water with MTZ (2.5 mM) under the same daily light/dark cycle.

### Flow cytometry and sorting

Flow cytometry and partial FACS analysis to isolate *siam*^+^, *mpo*^+^, *mpeg1*^+^, *lyzC*^+^ and *YFP*^+^ (NTR+) cells from various transgenic fish was performed beginning with isolation of the injured area fin tissue. Once isolated, this tissue was immediately placed in a tissue disassociation solution of 2 mg/ml collagenase (Sigma-Aldrich) and 0.3 mg/ml protease (type XIV, Sigma-Aldrich) in Hanks solution. The solution was moderately shaken at 30°C for 1 h with gentle trituration performed every 10 min with an 18 gauge needle. After 1 h, the solution was incubated for 5 min in 0.05% trypsin in PBS. Before flow cytometry, disassociated cells were washed in 2% (fetal bovine serum) FBS in cell disassociation solution. Disassociated cells from wild-type fish at an identical time point were used to set up the lower limit (background) of fluorescence in each experiment. For cleaved caspase 3 analysis, caspase 3 antibody (Sigma-Aldrich, AV00021; 1:200 in 2% FBS) was added to the suspension for 30 min on ice. After three successive washes with 2% FBS, fluorescently labeled secondary antibody was added (Alexa Fluor 647, Gt anti-mouse IgG; Life Technologies, A21236; 1:1000) for 20 min on ice. After three further washes (the last including 1:600 DAPI), the suspension was strained and read.

### Immunohistochemistry

Whole adult fin stumps (encompassing the entire fin plus 1-2 mm of the body girdle) were harvested and fixed in 4% formaldehyde in PBS overnight at 4°C. Tissue was then washed for 30 min at room temperature with 5% sucrose in PBS, followed by two washes for 1 h each in 5% sucrose in PBS, and an overnight wash in 30% sucrose in PBS at 4°C. After another overnight wash in a 1:1 ratio of 30% sucrose:100% O.C.T. compound (Tissue-Tek, VWR #25608-930) at 4°C, the tissue was embedded directly in 100% OCT in embedding wells and stored at −80°C before sectioning. Embedded tissue was sectioned in a cryostat and the entire dorsoventral span of the fin cut into 14 µm transverse sections and adhered to Superfrost Plus slides (VWR) overnight at 40°C.

Rabbit L-plastin antibody (a gift of Anna Huttenlocher; 1:300) or PCNA antibody (Sigma-Aldrich, P8825; 1:250) were added in antibody solution (0.5% Triton X-100, 5% goat serum, 0.2% BSA in PBS) for 2 h at room temperature in the dark. Slides were washed six times for 15 min each in antibody solution with gentle shaking, and goat anti-rabbit Alexa Fluor 647 secondary antibody (Life Technologies, A21244; 1:1000) added for 2 h at room temperature in the dark. After six more washes in antibody solution the slides were sealed with a coverslip with Prolong Gold Antifade Reagent (Life Technologies). EdU staining was performed according to Click-iT assays (Life Technologies, C10428). EdU was added 6 h prior to tissue extraction at 14 dpa.

For calcein-AM fluorochrome labeling, fish were immersed in 0.05% calcein-AM (Life Technologies, C3099) and rinsed for 10 min in fresh water. For analysis, the midpoint coordinates for all regenerated bone segments were manually identified and the corresponding calcein intensities were used to compute mean intensity (μ), standard deviation of intensity (σ), and coefficient of variation (σ/μ) for each fish.

### Quantitative RT-PCR

Total RNA was extracted from zebrafish fin regenerates using TRIZOL according to the manufacturer's protocol (Invitrogen). Tissue incorporating all new tissue as well as one or two bone rays proximal to the original cut site was extracted. Equal amounts of total RNA from each sample were reverse transcribed with Thermoscript reverse transcriptase (Invitrogen) using oligo(dT) and random hexamer primers. All levels were normalized to *β-actin* (*18S* levels were similar) and fold induction was calculated by setting control conditions to 1. Primers are listed in supplementary material Table S1.

## Supplementary Material

Supplementary Material
