# Supplementary material for: Macrophages modulate adult zebrafish tail fin regeneration
Source: Development. 2014 Jul;141(13):2581–91. doi: 10.1242/dev.098459 (PMC4067955; doi:10.1242/dev.098459)
Supplement: Supplementary Material [file supp_141_13_2581__index.html]

Macrophages modulate adult zebrafish tail fin regeneration — Supplementary Material 

# Macrophages modulate adult zebrafish tail fin regeneration

## DEV098459 Supplementary Material

**Files in this Data Supplement:**

- **Supplementary Material**
