# Supplementary material for: Macrophages modulate adult zebrafish tail fin regeneration
Source: Development. 2014 Jul;141(13):2581–91. doi: 10.1242/dev.098459 (PMC4067955; doi:10.1242/dev.098459)
Supplement: Supplementary Material [file supp_141.13.2581_DEV098459.pdf]

Fig. S1. Adult neutrophils are characterized by lysozyme C (lyzC) and myeloperoxidase (MPO) expression. (**A-F**) Amputated fins from Tg(*lyzC*:dsRed) and Tg(*MPO*:GFP) fish; (D-F) are insets of (A-C). (**G**,**H**) Tg(*mpeg1*:YFP; *lyzC*:dsRed) body midline indicating that macrophages (mpeg1+) do not overlap with lyzC+ cells.

Fig. S2. Neutrophils migrate through circulation to wound site and adhere to endothelium at injury. Time sequence of a neutrophil in Tg(*mpo*:GFP) fish circulating through inter-ray caudal fin vein after 4 hpa, slowing, and adhering to endothelial walls, and beginning to extravagate outward to interstitial tissue at wound site. Red arrows refer to neutrophil undergoing rolling adhesion.

Fig. S3. Neutrophil recruitment in regenerating caudal fins in Tg(*lyzC*:dsRed) fish. Quantification of neutrophil number near the amputated fin edge using flow cytometry and TFI methodology. Pooled amputated fins from Tg(*lyzC*:dsRed) adult zebrafish at 1 dpa, 3 dpa, 6 dpa, and 9 dpa were disassociated and numbers of *lyz*C+ cells relative to total cells were quantified from flow cytometry. Plot of cell density per injured area over multiple time points by TFI (y-axis) and flow cytometry (x-axis). R^2= .99Samples were pooled and cell numbers averaged over successive experiments.

Fig. S4. Neutrophil deficiency near amputation plane does not affect regeneration. (A) Experimental scheme. Adult Tg(*mpo*:GFP) fish were incubated in diphenyleneiodonium chloride (DPI) at 30 µM for 12 h before caudal fins were amputated with a distal resection. DPI was added to fish water daily for 3 dpa. After 3 dpa, fish were incubated in water without DPI for the rest of the experiment (10 dpa). Yellow arrowheads represent cut plane. (**B**) Neutrophil (mpo+) density near the injury area was reduced with DPI treatment. Representative images of mpo+ cells near the amputation plane at 2 dpa. Scale bar = 100 µm. (**C**) Rate of fin regeneration was not significantly different between DPI-treated (DPI) (N=7) and untreated (DMSO) (N=6) fish for duration of experiment. Error bars represent the s.e.m. Data representative of two separate experiments.

Fig. S5. Tg(*mpeg1*:NTR-YFP) fish line affords inducible systemic ablation of macrophages. (A) Schematic of a new inducible macrophage ablation fish line Tg(mpeg1:NTR-eYFP) made using NTR/MTZ technology. Briefly, fish expressing the *mpeg1* promoter ubiquitously express a fusion NTR-YFP protein. Addition of metronidazole (MTZ) in fish water results in catalytic electron transfer and activation of Met in cells expressing NTR fusion protein. Activated Met promotes DNA breaks and ultimately cell death. Representative image of *mpeg1*+ cell (macrophage) in upper panel. Scale bar = 10 µm. (**B**) Macrophages (*mpeg1*+) are easily visualized throughout the caudal fin in Tg(*mpeg1*:NTR-eYFP) fish as green YFP+ fluorescent cells (upper panels). Subsequent incubation in 2.5 mM MTZ for 12h and 36h results in dissipation of this green signal and significant reduction of overall numbers of macrophages throughout the fish, including the caudal fin (lower panels). Scale bar = 200 µm. (**C**) MTZ-induced cell ablation in Tg(mpeg1:NTR-eYFP) fish as assessed in uninjured +/- MTZ treated fins via flow cytometry shows an 80-90% reduction in eYFP+ cells post-MTZ treatment (2d). Flow cytometry was conducted on pooled samples (NTR+MTZ, N=8-10; NTR-MTZ, N=8-10). Data is representative of 3 separate experiments. Error bars are S.E.M. (**D**) Quantification of macrophage cell number near the amputation planes for both the proximal and distal cuts (N=7) from uncut to 14 dpa in Tg(*mpeg1*:NTR-eYFP) fish. Data is representative of 2 separate experiments.

Fig. S6. Macrophage ablation is inducible and systemic in Tg(*mpeg1*:NTR-YFP) fish. (A) Macrophages are easily visualized throughout the pectoral fin and ocular area in Tg(*mpeg1*:NTR-eYFP) fish and addition of MTZ (for 2d) visually decreases macrophage number. Scale bar = 60 µm. (**B**) Macrophage ablation is reversible by MTZ washout. Incubation of transgenic fish in MTZ for 36 h ablates most macrophages, and subsequent incubation in non-drug fish water for >2d results in gradual restoration of overall numbers of macrophages throughout the fish, including the caudal fin. (**C**) Close-up images of mepg1+ and YFP+ cells in Tg(*mpeg1*:mCherry) and Tg(*mpeg1*:NTR-eYFP) fish in the caudal fin. Scale bar = 20 µm.

Fig. S7. MTZ treatment does not affect overall cell apoptosis or inflammation in wild-type regenerating caudal fins. (A) Flow cytometry profile of caspase-3 positive cells in wild-type adult zebrafish fins treated with or without MTZ. MTZ treatment (for 7d) did not affect total numbers of apoptotic cells in tail fins. Samples were

pooled from 5-7 zebrafish tail fins per condition, representative of two experiments. (**B**) No difference in neutrophil accumulation in the injury area after fin resection was found between Tg(*lyzC:*dsRed) fish treated with MTZ (N=6) compared to untreated fish (N=6). (**C**) No difference in macrophage accumulation in the injury area after fin resection was found between Tg(*mpeg1:*mCherry) fish treated with MTZ (N=7) compared to untreated fish (N=6). Error bars represent the s.e.m.

Fig. S8. Macrophage depletion does not affect neutrophil accumulation near injury in regenerating caudal fins. Quantification of neutrophil accumulation in injury area of Tg(*mpeg1*:NTR-eYFP;*lyzC*:dsRed) and Tg(*lyzC*:dsRed) fish treated continuously with MTZ after fin resection. No significant difference between macrophage-depleted fish (NTR+MTZ, N=8) and wild-type fish (WT+MTZ, N=9) was found. Error bars represent the s.e.m. data representative of 2 separate experiments.

Fig. S9. Macrophage depletion is maintained in adult caudal fins through 14 dpa. (**A,B**) Transverse and flat sections of Tg(mpeg1:NTR-YFP) amputated caudal fins at 7 dpa and 14 dpa with continuous MTZ treatment (NTR+MTZ) and without (NTR-MTZ). (**C**) Flow cytometry of pooled 14 dpa fins gated for YFP+ (macrophages) and Dapi-(live cells) in NTR-MTZ and NTR+MTZ fish. (**D**) Quantification of flow cytometry of Dapi-YFP+ population as a percentage of total events for 14 dpa pooled fins Error bars represent the s.e.m. data representative of 2 separate experiments. (**E**) Representative fin images (YFP) for temporally ablated fish at 3 dpa (where either MTZ was washed out or MTZ added for the first time) and 5 dpa. Scale bar = 200 µm.

Fig. S10. Macrophage depletion negatively affects larval fin regeneration. (A) Macrophages were continuously ablated before and after larval fin resection (up to 5 dpa) using the macrophage ablation fish line Tg(*mpeg1*:NTR-eYFP). Representative YFP images of macrophages in both uncut and 5 dpa transgenic larvae. Red arrows indicate individual macrophages. Blue dotted line indicates original cut site. **(B**) Representative whole fin hybrid DIC/YFP images of wild-type fish or Tg(*mpeg1*:NTR-eYFP) fish undergoing continuous macrophage ablation after resection. Red arrows point toward original cut line. (**C**) Quantification of regenerated tissue as a percentage of original fin area for macrophage-ablated fish (NTR+MTZ, N=9), wild type fish (WT+MTZ, N=11) and transgenic fish controls (NTR-MTZ, N=14). Full regeneration to the original fin area is identical to 100% regeneration. Numbers are compiled and averaged over two separate experiments using identical conditions. *P (5dpa) = 0.0314 (two-tailed).

Fig. S11. Extended macrophage depletion affects regenerative and cytokine gene expression. Gene expression levels of pooled blastema fin tissue (N>5) as assessed by quantitative RT-PCR for amputated WT and Tg(*mpeg1*:NTR-eYFP) fins at 1 dpa or 4 dpa with or without continuous MTZ treatment. Levels were normalized to fold over NTR-MTZ control and data averaged over 2 separate experiments and 3 technical replicates per experiment/condition.

Fig. S12. Wnt signaling inhibition delays neutrophil resolution in amputated larval fins. (**A**) Representative images (24 hpa) of a loss-of-function Wnt/β-catenin signaling line Tg(*hsDKK1*:GFP) crossed to a Tg(*lyzC*:dsRed) line to detail a regenerative timecourse of neutrophil accumulation in larval fins near the injury site. Fins on wild-type (WT) or loss-of-function Wnt/β-catenin signaling fish (hsDKK) were resected, and heat shocked once daily (hsWT, hsDKK) for 80 hpa. (**B**) Quantification of neutrophil accumulation in injury area after fin amputation for hsDKK and WT fish indicating increased cell number in DKK1-overxpressing fins at time points from 16 hpa through 60 hpa. (C) Quantification of neutrophil accumulation in injury area after fin amputation in gain-of-function Wnt/β-catenin signaling line Tg(*hsWnt8a*:GFP) crossed to a Tg(*lyzC*:dsRed) line. Unlike loss-of-function Wnt/β-catenin signaling fish (hsDKK1), Wnt8a-overexpressing larvae show no significant difference in macrophage number with hsWT. Data are representative of three independent experiments with at least 6-8 fish per timepoint. Error bars represent the s.e.m. * vs hsWT; P(16 hpa)=0.0140, P(20 dpa) = 0.0125, P(28 dpa) = 0.0399.

Fig. S13. Modulation of Wnt signaling affects cytokine phenotype of macrophages in regenerating fins. Gene expression levels of sorted macrophages (N>5) as assessed by quantitative RT-PCR for amputated WT and a loss-of-function Wnt/β-catenin signaling Tg(*hsDKK1*:GFP) fins at 3 dpa or 7 dpa with only one heat shock (pulse) at 3

and 7 dpa. Macrophages were sorted 12h after heat shock after these time points. Levels were normalized to fold over non-heat shock WT control and data averaged over 2 separate experiments and 3 technical replicates per experiment/condition.

Fig. S14. Delayed modulation of Wnt signaling affects migration of macrophages in regenerating fins. Tg(*hsDKK1*:GFP) and WT fish were heat shocked daily beginning 3 dpa (A,B) or 5 dpa (C,D) and macrophage presence in regenerating tissue quantified (B,D). (A) Representative images of macrophages at 7 dpa after heat shock beginning at 3 dpa. (B) Macrophage presence was lower in Tg(*hsDKK1*:GFP) fish compared to WT fish from 7 dpa and on. (C) Representative images of macrophages at 10 dpa after heat shock beginning at 5 dpa. Macrophage presence was lower in Tg(*hsDKK1*:GFP) fish compared to WT fish from 7 dpa and on, although not to the same extent as heat shocking at 3 dpa.

Movie 1. Short movie in distal caudal fin of Tg(mpo:GFP) fish 6 h after resection showing real-time tracking of neutrophils through vasculature and past injury site. Note occasional neutrophilic behavior indicative of rolling and extravasation into the interray region.

Figure S1.

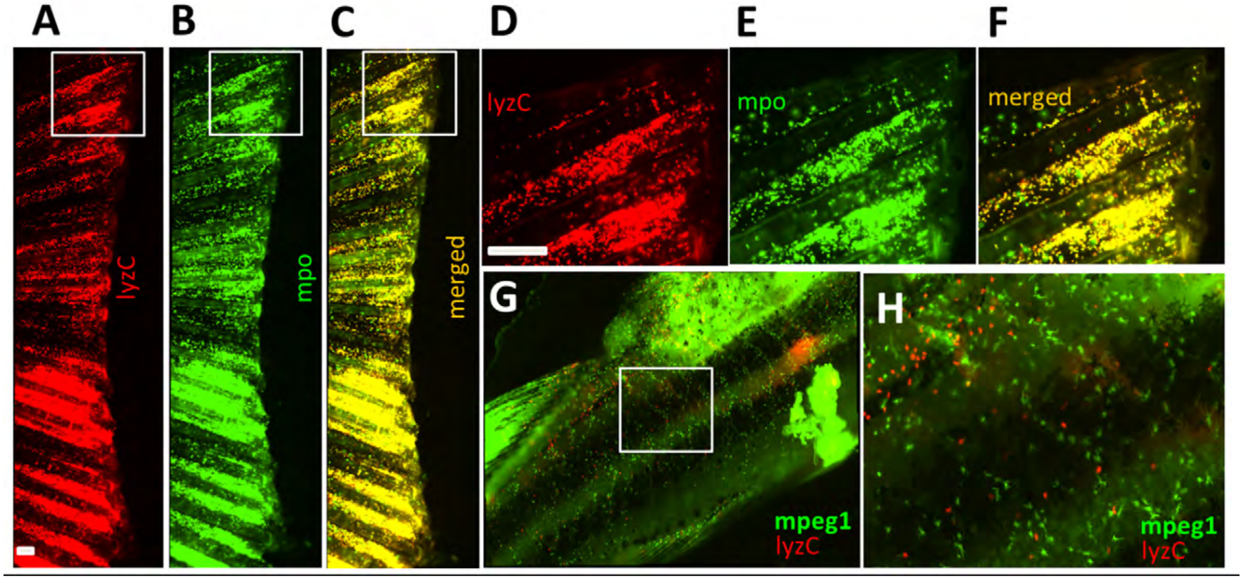

Figure S2.

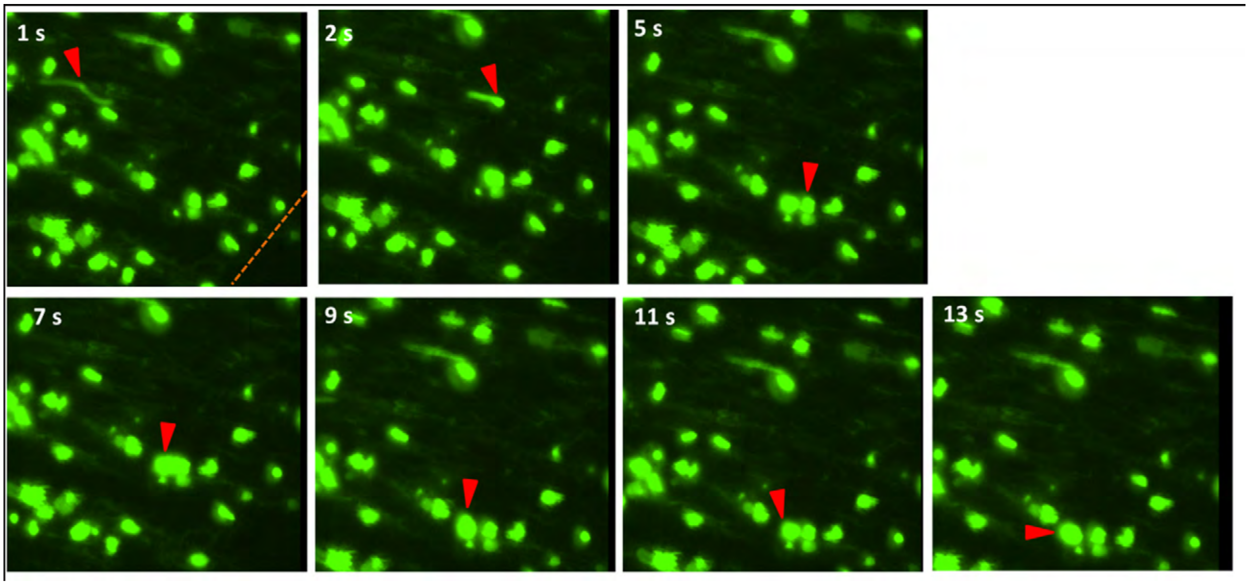

Figure S3.

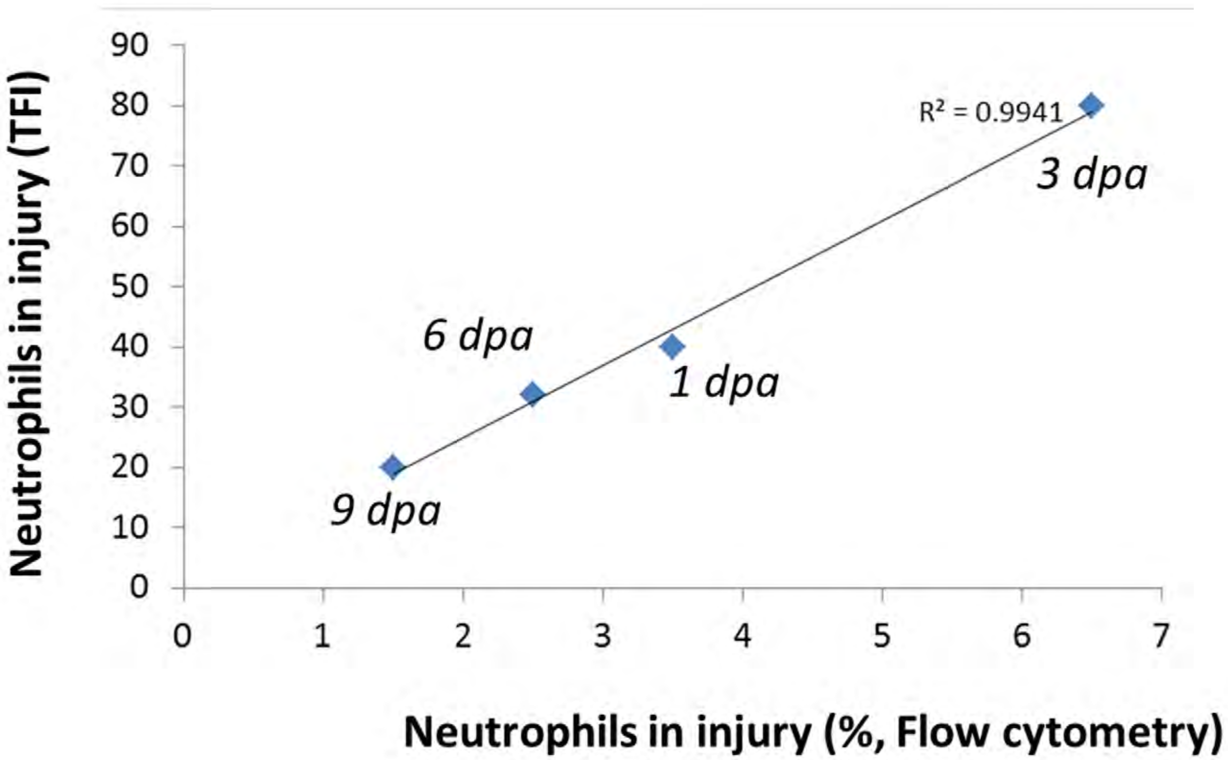

Neutrophils in injury (TFI)
90
80
70
60
50
40
30
20
10
0
R² = 0.9941
3 dpa
6 dpa
1 dpa
9 dpa
0
1
2
3
4
5
6
7
Neutrophils in injury (%, Flow cytometry)

Figure S4.

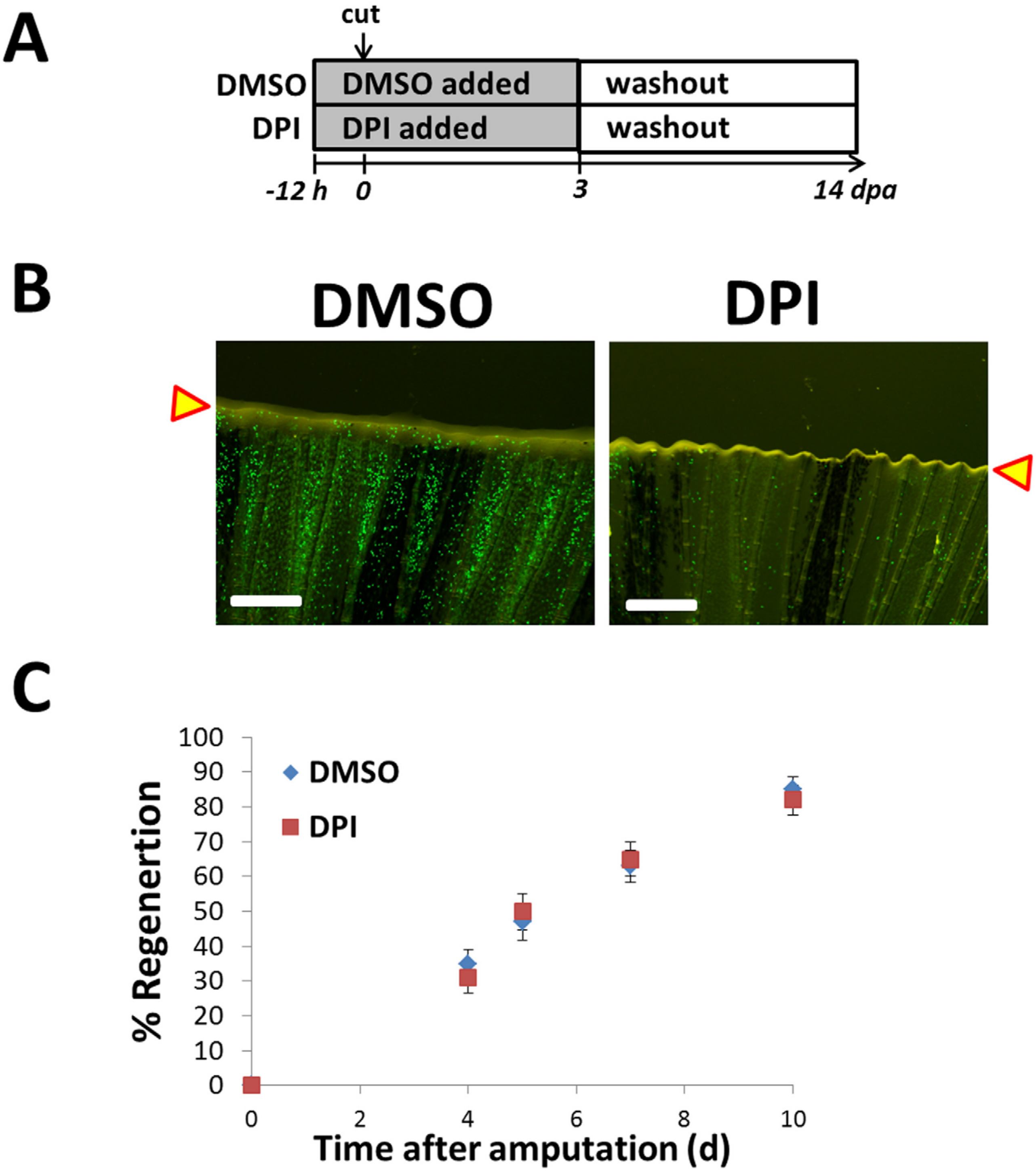
A
cut
DMSO
DPI
DMSO added
DPI added
washout
washout
-12 h
0
3
14 dpa
B
DMSO
DPI
C
DMSO
DPI
% Regenertion
Time after amputation (d)
0
10
20
30
40
50
60
70
80
90
100
0
2
4
6
8
10

Figure S5.

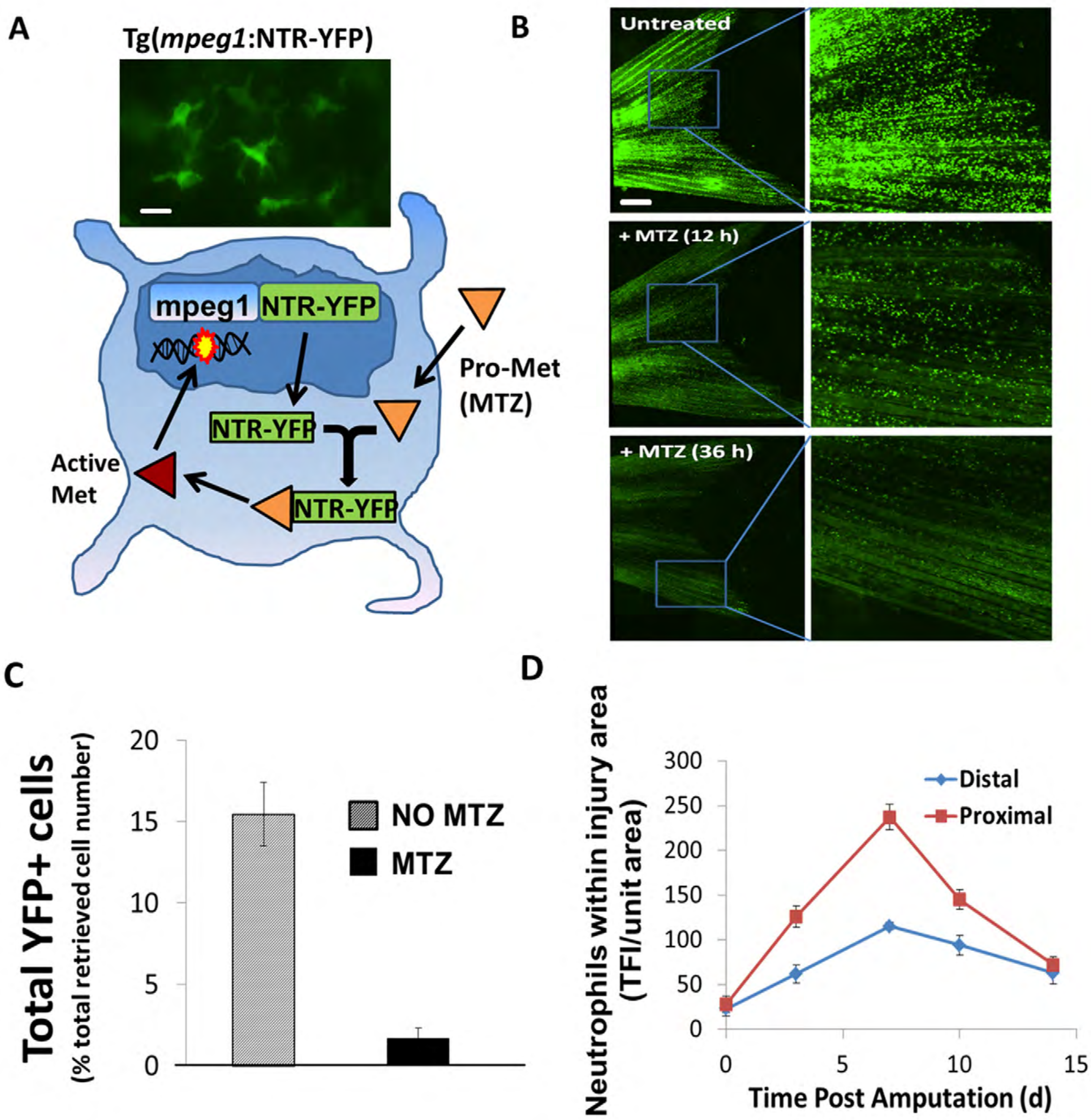

A
Tg(*mpeg1*:NTR-YFP)
mpeg1
NTR-YFP
NTR-YFP
NTR-YFP
Pro-Met
(MTZ)
Active
Met
B
Untreated
+ MTZ (12 h)
+ MTZ (36 h)
C
Total YFP+ cells
(% total retrieved cell number)
20
15
10
5
0
NO MTZ
MTZ
D
Neutrophils within injury area
(TFI/unit area)
300
250
200
150
100
50
0
Distal
Proximal
0
5
10
15
Time Post Amputation (d)

Figure S6.

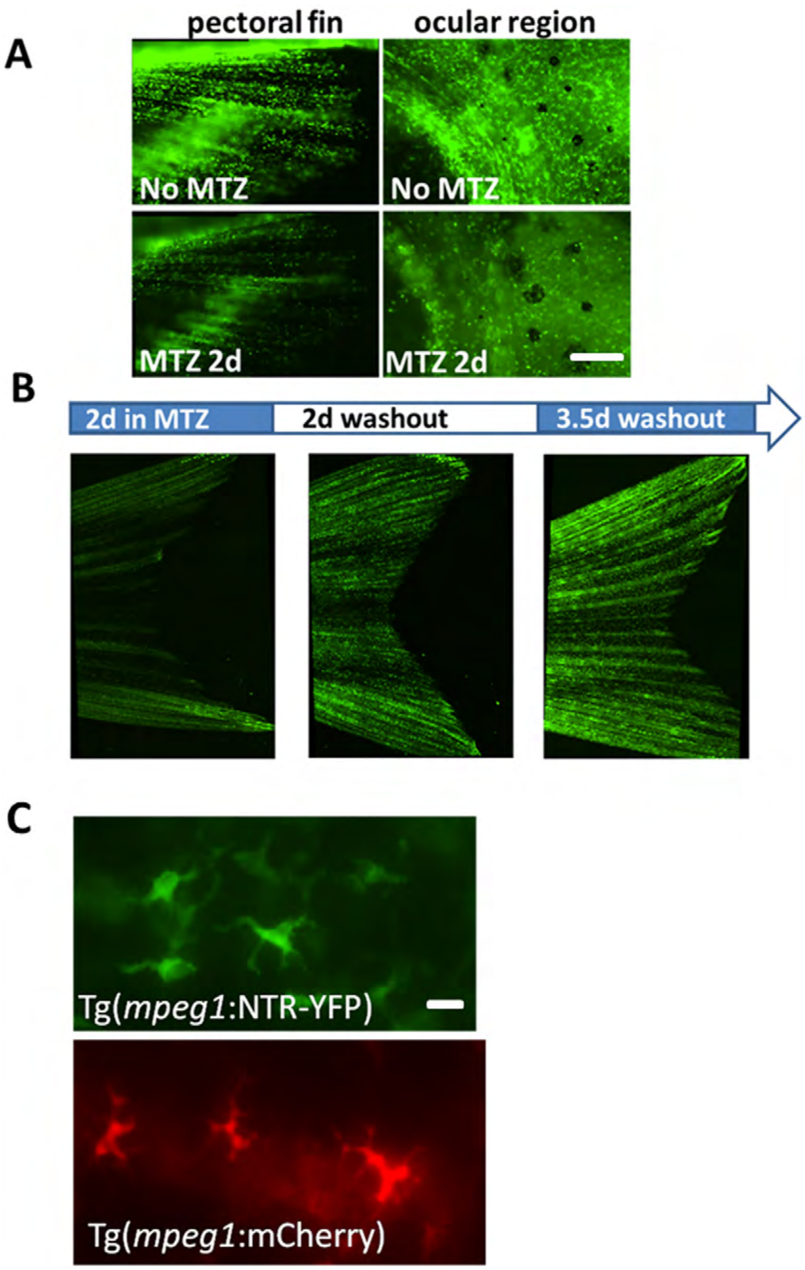

A
pectoral fin
ocular region
No MTZ
No MTZ
MTZ 2d
MTZ 2d
B
2d in MTZ
2d washout
3.5d washout
C
Tg(mpeg1:NTR-YFP)
Tg(mpeg1:mCherry)

Figure S7.

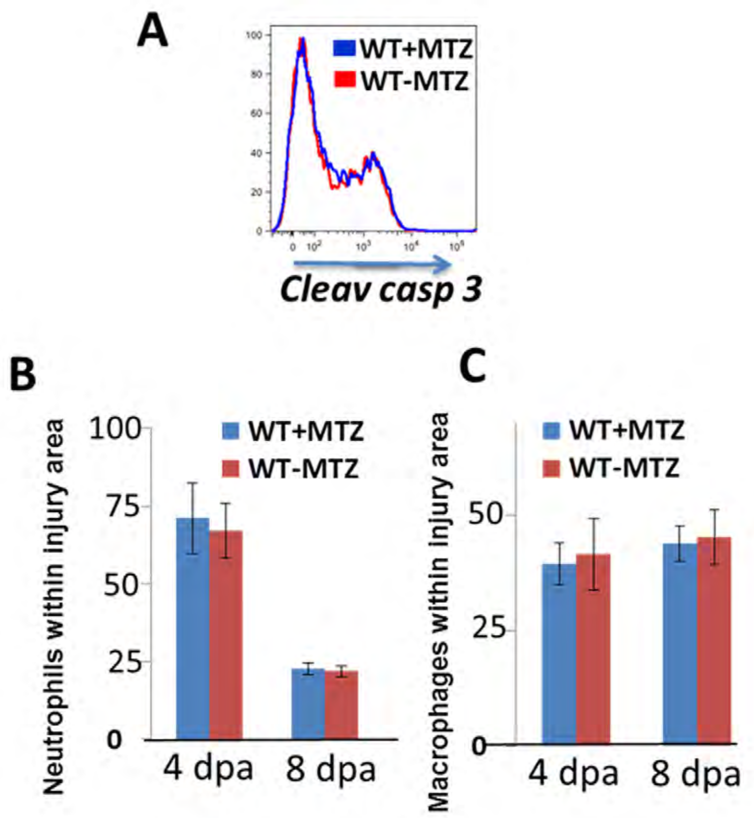

Figure S8.

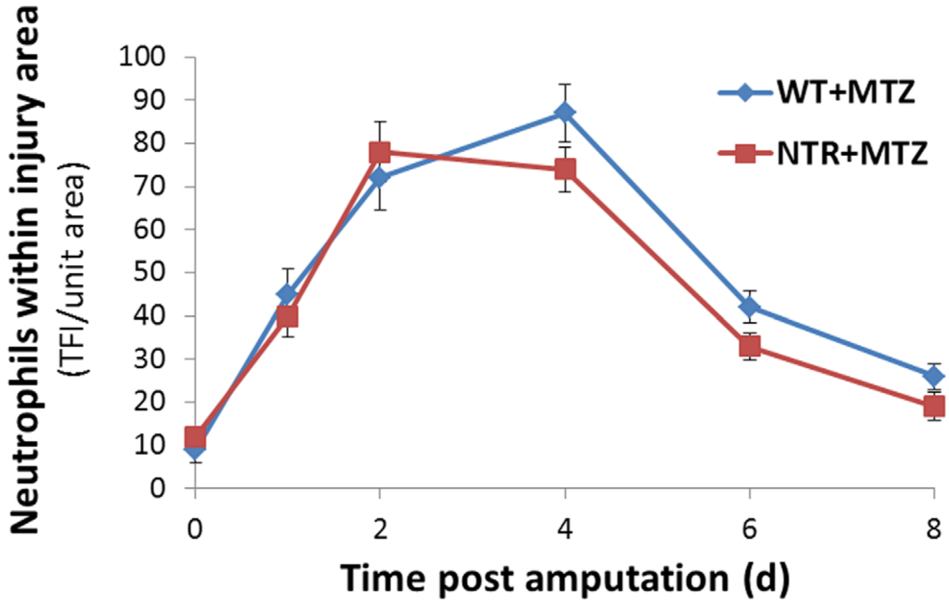

Figure S9.

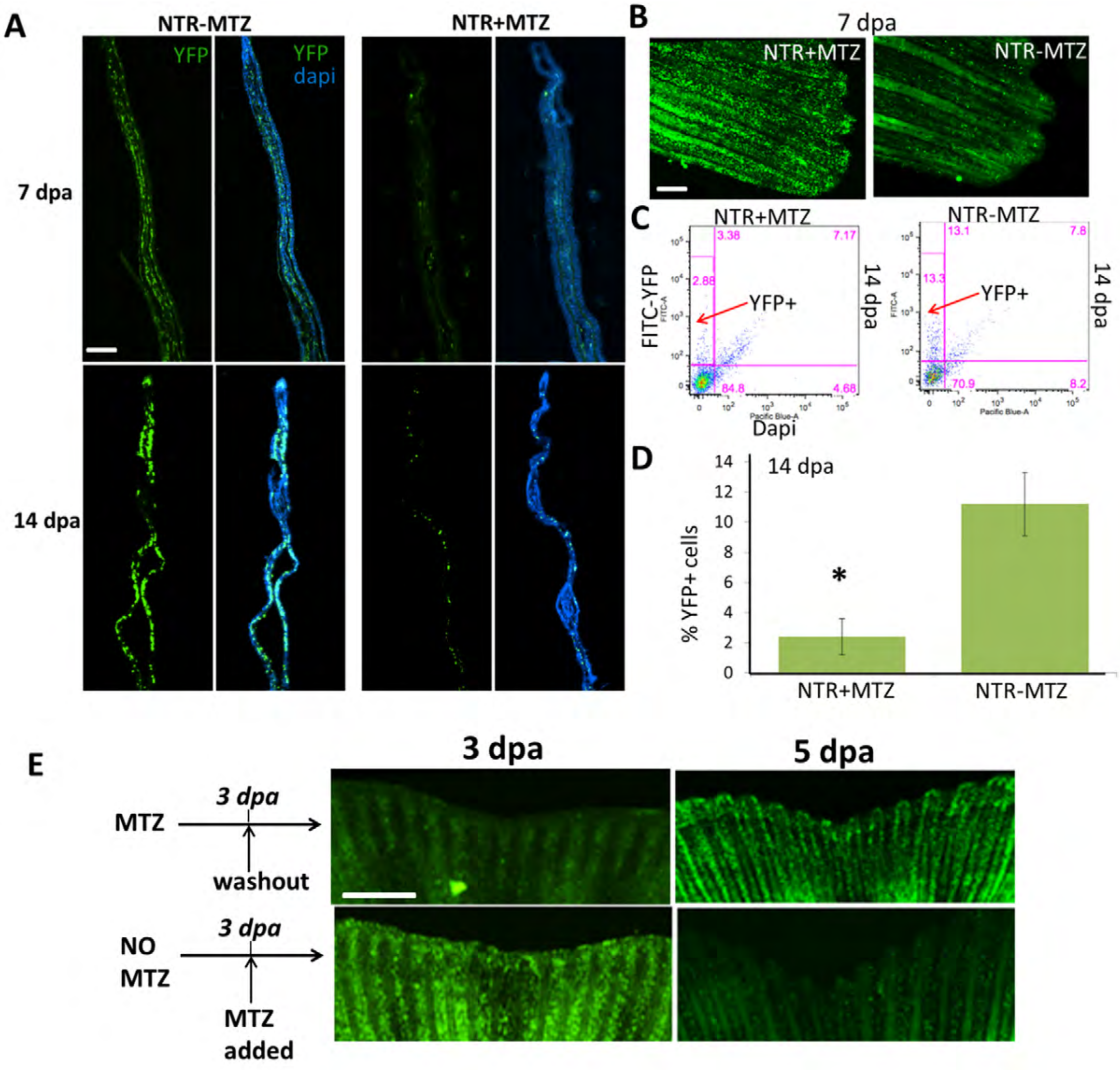
A
NTR-MTZ
NTR+MTZ
YFP
YFP
dapi
7 dpa
14 dpa
B
7 dpa
NTR+MTZ
NTR-MTZ
C
NTR+MTZ
NTR-MTZ
FITC-YFP
YFP+
14 dpa
Dapi
3.38
7.17
2.88
84.8
4.68
13.1
7.8
13.3
70.9
8.2
D
14 dpa
% YFP+ cells
*
NTR+MTZ
NTR-MTZ
E
3 dpa
5 dpa
MTZ
3 dpa
washout
NO
MTZ
3 dpa
MTZ
added

Figure S10.

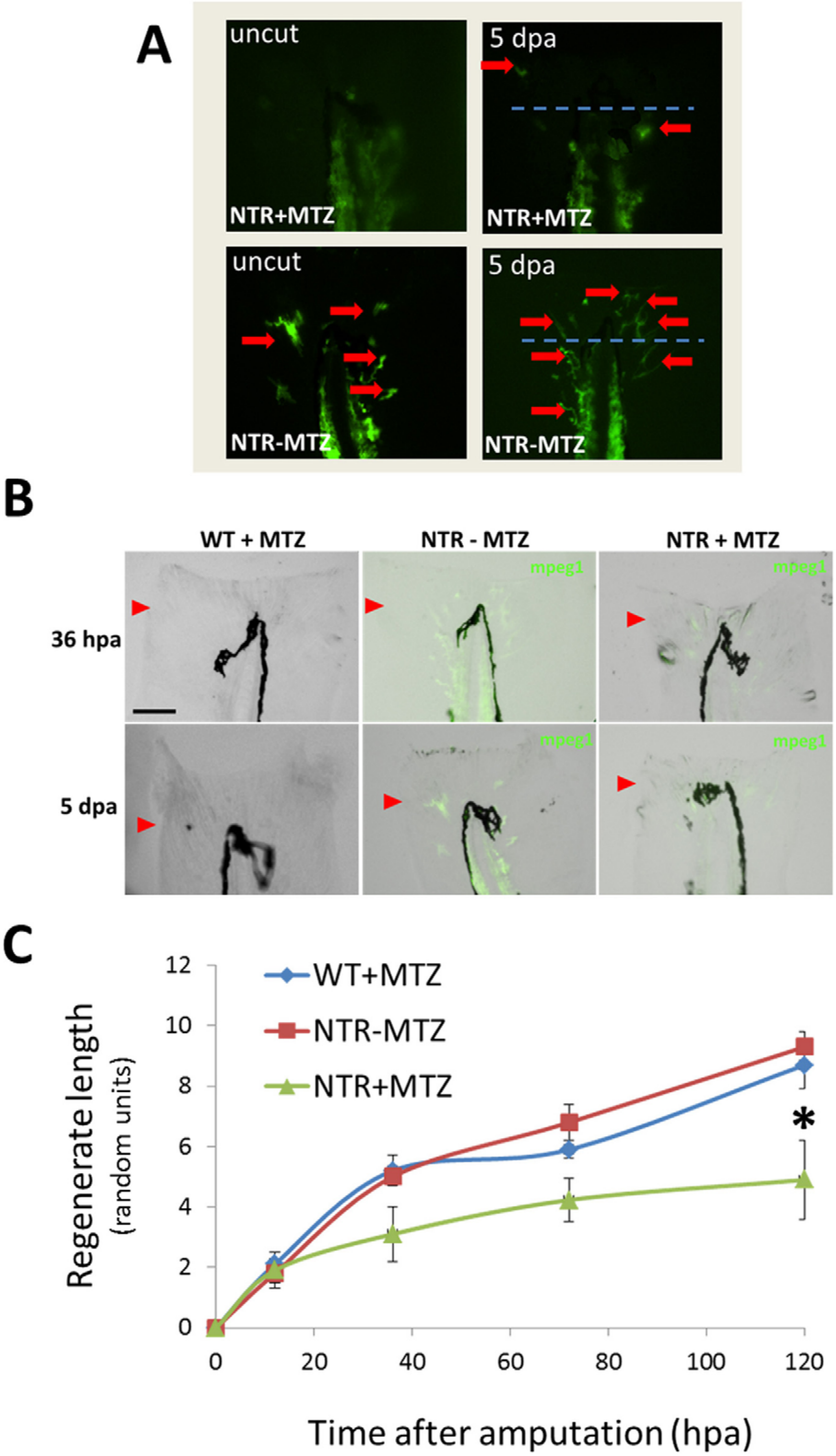
A
uncut
NTR+MTZ
5 dpa
NTR+MTZ
uncut
NTR-MTZ
5 dpa
NTR-MTZ
B
WT + MTZ
NTR - MTZ
NTR + MTZ
mpeg1
36 hpa
5 dpa
C
WT+MTZ
NTR-MTZ
NTR+MTZ
Regenerate length
(random units)
Time after amputation (hpa)
*
0
2
4
6
8
10
12
0
20
40
60
80
100
120

Figure S11.

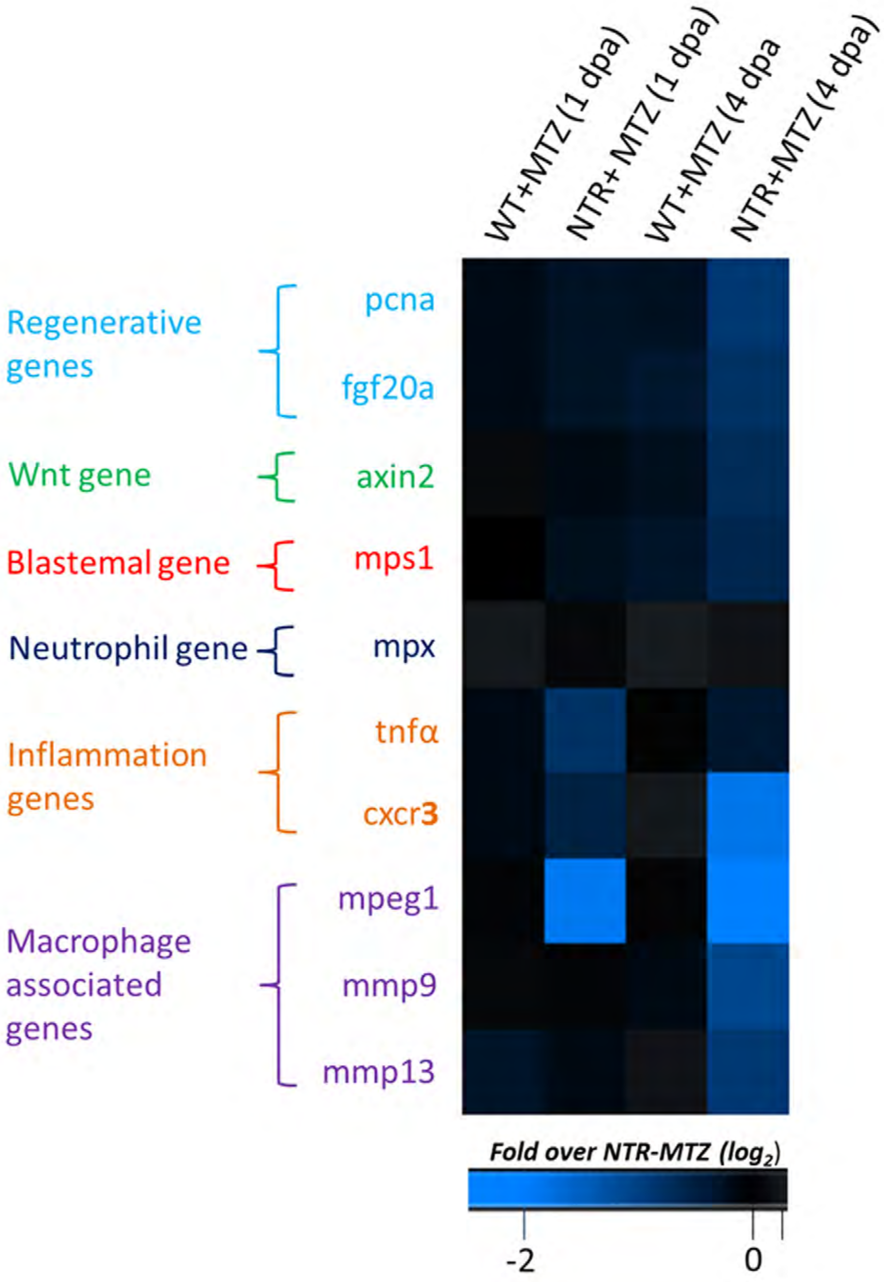
WT+MTZ (1 dpa)
NTR+ MTZ (1 dpa)
WT+MTZ (4 dpa
NTR+MTZ (4 dpa)
Regenerative genes
pcna
fgf20a
Wnt gene
axin2
Blastemal gene
mps1
Neutrophil gene
mpx
Inflammation genes
tnfα
cxcr3
Macrophage associated genes
mpeg1
mmp9
mmp13
Fold over NTR-MTZ (log2)
-2
0

Figure S12.

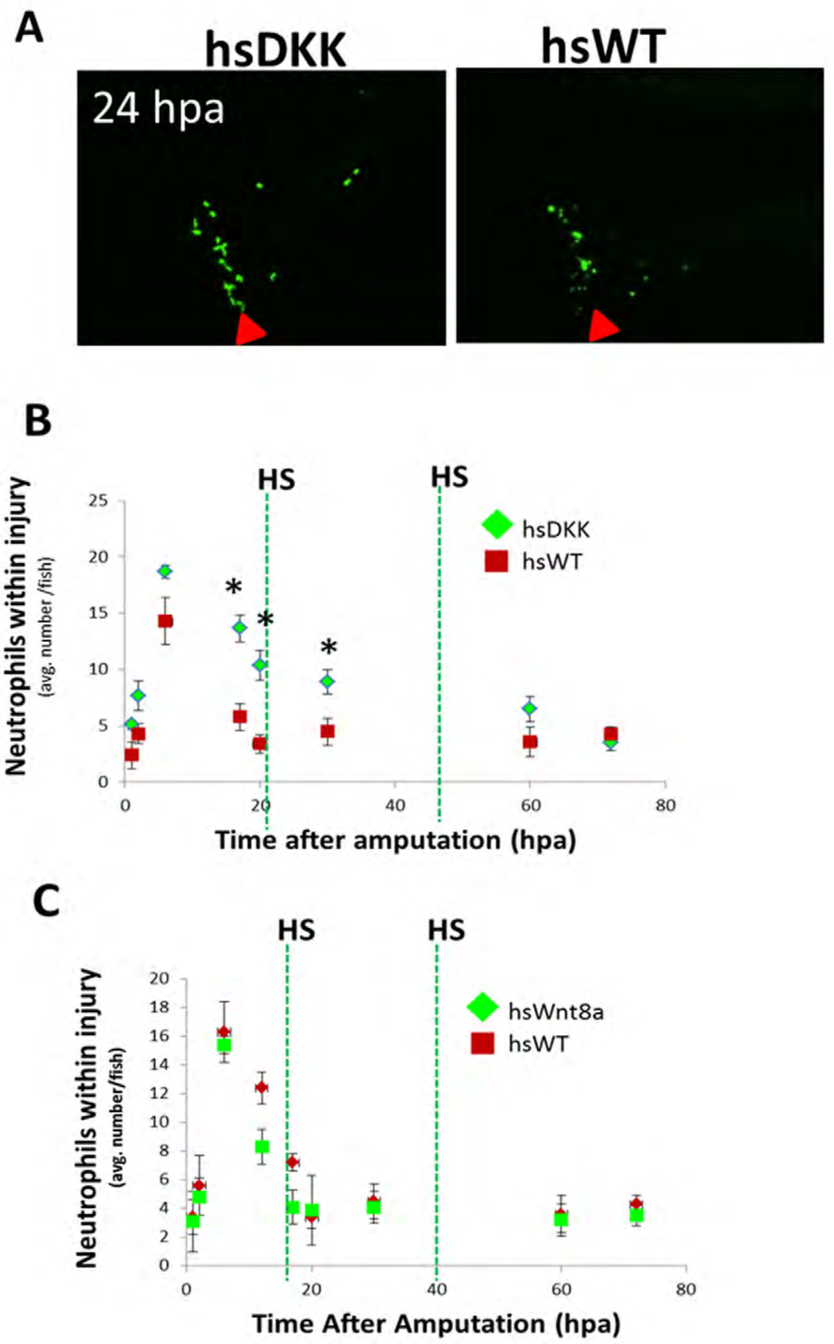
A
hsDKK
hsWT
24 hpa
B
HS
HS
Neutrophils within injury
(avg. number /fish)
hsDKK
hsWT
*
*
*
25
20
15
10
5
0
0
20
40
60
80
Time after amputation (hpa)
C
HS
HS
Neutrophils within injury
(avg. number/fish)
hsWnt8a
hsWT
20
18
16
14
12
10
8
6
4
2
0
0
20
40
60
80
Time After Amputation (hpa)

Figure S13.

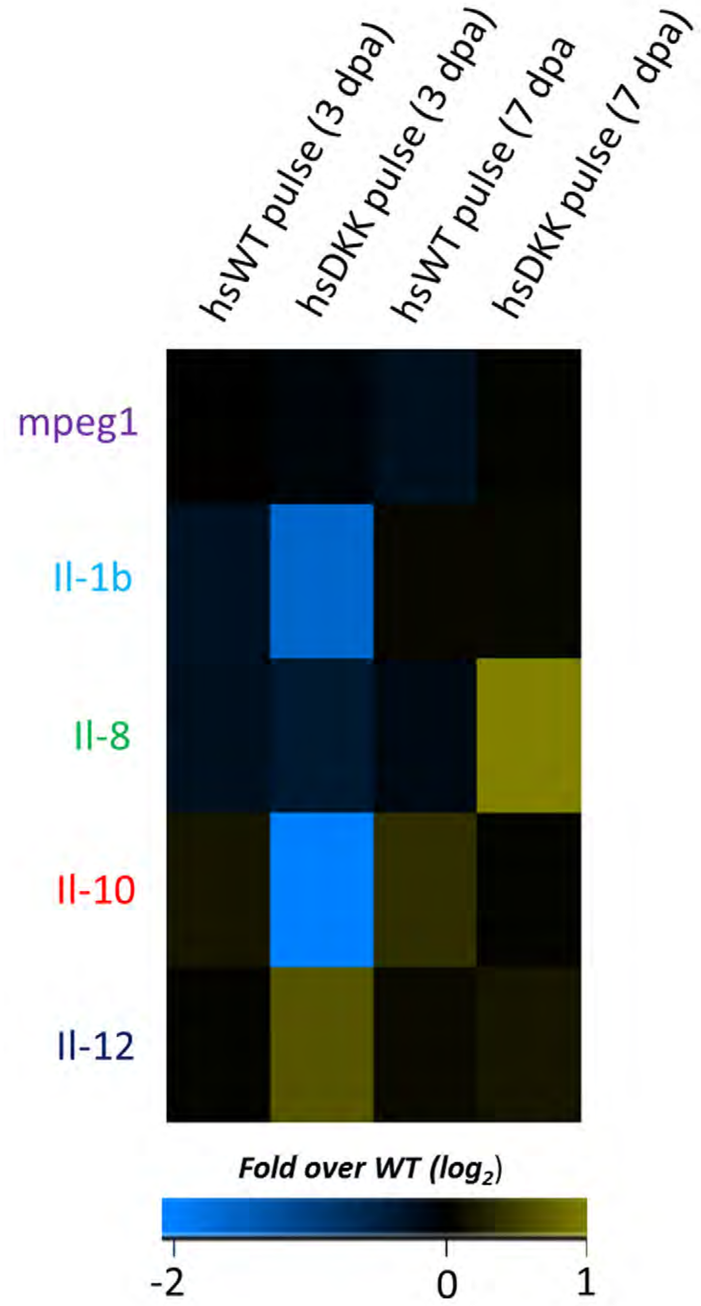
hsWT pulse (3 dpa)
hsDKK pulse (3 dpa)
hsWT pulse (7 dpa
hsDKK pulse (7 dpa)
mpeg1
Il-1b
Il-8
Il-10
Il-12
Fold over WT (log2)
-2
0
1

Table S1. Primers used for qRT-PCR

Gene	Forward Primer	Reverse Primer
pcna	CCCGATTGTGACCCTCTAAA	TTGGAATGAGCAFGTTGGACA
axin2	GGAAGAGGGTGAGACGACA	AATGGGACGCACTGCTACT
mpx	TCGTAGTTTGGGCTTTGTGA	TAGCCTCAAACGGGAAAAAG
ilb	TTGTGGGAGACAGACAGTGC	GATTGGGGTTTGATGTGCTT
tnfa	ACAAGGCAATTTCACTTCCA	AGCTGATGTGCAAAGACACC
il8	TGTTTTCCTGGCATTTCTGACC	TTTACAGTGTGGGCTTGGAGGG
il10	ACGCTTCTTCTTTGCGACTG	CACCATATCCCGCTTGAGTT
il12	AGCAGGACTTGTTTGCTGGT	TCCACTGCGCTGAAGTTAGA
fgf20a	CAGCTTCTCTCACGGCCTTGG	AAAGCTCAGGAACTCGCTCTG
mmp13	ATGGTGCAAGGCTATCCCAAGAGT	GCCTGTTGTTGGAGCCAAACTCAA
mps1	ACTCGCAGGTCGGAACTCTG	CCACACGTCCCCTTTAGCAC
mmp9	AACCACCGCAGACTATGACAAGGA	GTGCTTCATTGCTGTTCCCGTCAA
mpeg1	CCCACCAAGTGAAAGAGG	GTGTTTGATTGTTTTCAATGG
cxcr3	ATGGACAACTCAACAACAGC	GTAAGCCACAGGTGCAAAG
beta-actin	CTTGCGGTATCCACGAGAC	GCGCCATACAGAGCAGAA

Figure S14.

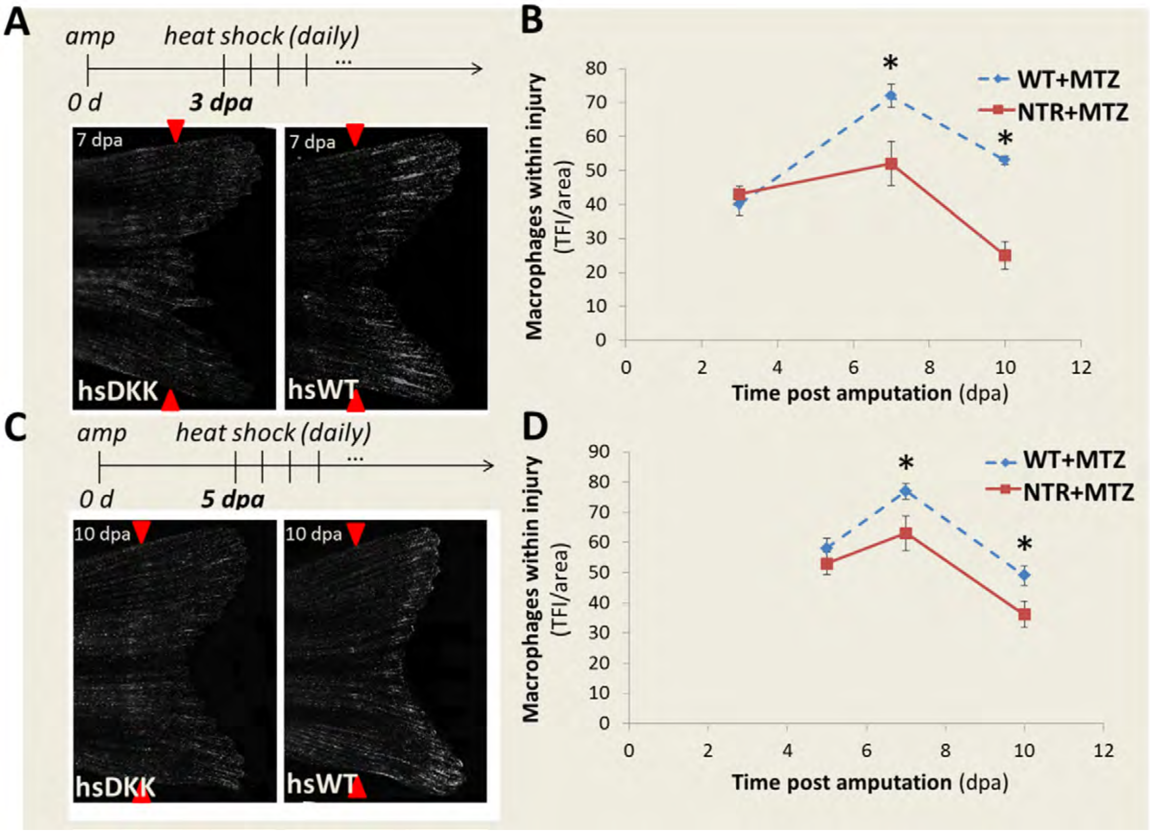

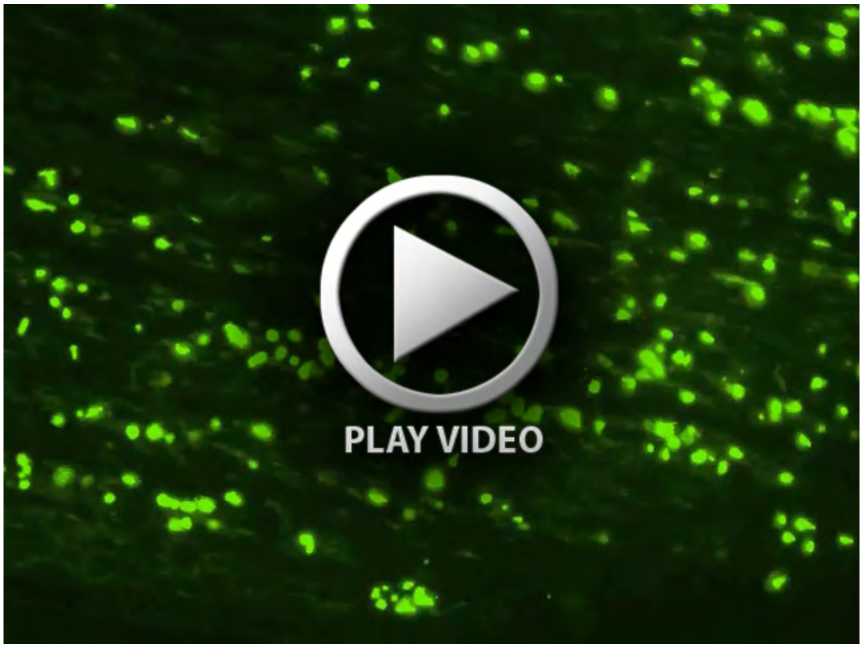

Movie 1
